# Dietary patterns, genetic predisposition, and risk of cholelithiasis: a large-scale prospective cohort study

**DOI:** 10.3389/fnut.2024.1469789

**Published:** 2024-09-27

**Authors:** Kecheng Jin, Ningning Mi, Wangping He, Ruyang Zhong, Boru Jin, Zhen Liu, Chunlu Dong, Yanyan Lin, Ping Yue, Bin Xia, Qiangsheng He, Jinqiu Yuan, Wenbo Meng

**Affiliations:** ^1^The First School of Clinical Medicine, Lanzhou University, Lanzhou, China; ^2^Department of General Surgery, The First Hospital of Lanzhou University, Lanzhou, China; ^3^Clinical Research Center, Big Data Center, The Seventh Affiliated Hospital, Sun Yat-sen University, Shenzhen, China

**Keywords:** dietary patterns, genetic risk, cholelithiasis, UK Biobank, gene-diet interactions, dietary intervention

## Abstract

**Background:**

Limited epidemiological evidence exists concerning the impact of healthy dietary patterns on reducing the risk of cholelithiasis. We aimed to examine the association of seven established dietary patterns with subsequent cholelithiasis risk and whether this association was modified by genetic risk.

**Methods:**

We conducted a prospective cohort study from the UK Biobank, including 155,323 participants initially free of cholelithiasis and cholecystectomy. Dietary patterns were assessed using a validated food frequency questionnaire (Oxford WebQ), covering Mediterranean Diet Score (MED), alternate Mediterranean Diet Score(aMED), overall Plant-based Diet Index (PDI), healthy Plant-based Diet Index (hPDI), unhealthy Plant-based Diet Index (uPDI), Healthy Eating Index 2015 (HEI-2015) and EAT-lancet Score. Genetic risk was quantified and stratified by a polygenic risk score (PRS) incorporating 13 known cholelithiasis-associated loci. Cox proportional hazards regression was employed to estimate the association between dietary patterns, PRS, and cholelithiasis incidence, adjusting for potential confounders.

**Results:**

During a median follow-up of 13.3 years, 5,056 cases of cholelithiasis were identified. After adjusting for potential confounders, adherence to aMED and HEI-2015 dietary patterns reduced cholelithiasis risk by 10% (HR: 0.90; 95%CI: 0.83–0.98) and 11% (HR: 0.89; 95%CI: 0.82–0.96), respectively. A significant decrease in cholelithiasis risk was observed across PRS quintiles, low PRS was associated with a 16% reduced risk (HR: 0.84; 95%CI: 0.77–0.92). Participants with both high dietary scores and low genetic risk had the lowest cholelithiasis risk, with an HR of 0.76 (95%CI: 0.64–0.91) for aMED and 0.73 (95%CI: 0.61–0.88) for HEI-2015.

**Conclusion:**

Higher adherence to aMED and HEI-2015 might significantly decrease the risk of cholelithiasis, irrespective of genetic risk. Our results highlighted the potential of diet intervention for cholelithiasis prevention in the general population.

## 1 Introduction

Cholelithiasis is a common gastrointestinal disease with an average prevalence of 10 ~ 20% among the global adult population (1), which likely results from a complex interaction of genetic susceptibility and environmental factors (1–3). Diet, among these factors, has been highlighted as one of the most influential environmental contributors and is associated with cholelithiasis (4). Dietary patterns that consider multiple foods and components together provide a comprehensive understanding of dietary exposures. Specific dietary patterns, such as those high in refined carbohydrates and saturated fats, have been linked to increased cholesterol saturation in bile, which is a key factor in gallstone formation. Based on previous literature and dietary guidelines (5–10), we identified seven dietary pattern scores. Investigating the impacts of dietary patterns on cholelithiasis in a large cohort can enhance our understanding of the role of diet in cholelithiasis development.

With the development of genome-wide association studies (GWAS), genetic susceptibility loci for cholelithiasis have been discovered in prior studies (11, 12). By aggregating these loci, polygenic risk scores (PRS) can be constructed to effectively estimate the genetic risk of cholelithiasis. However, the predictive performance of PRS varies among individuals depending on their environmental exposures (11, 12), suggesting that the genetic risk of cholelithiasis could be offset by non-heritable factors. Identifying these factors, particularly dietary patterns, is crucial for enabling individuals with high genetic risk to benefit from healthy diets. To our knowledge, no studies have investigated the individual and combined associations of genetic variations and dietary patterns with cholelithiasis risk. Furthermore, the extent to which healthy dietary patterns can offset cholelithiasis risk among individuals with different genetic predispositions remains unknown.

To address these issues, we assessed the relationships between seven distinct dietary patterns [Mediterranean Diet Score (MED), alternate Mediterranean Diet Score (aMED), overall Plant-based Diet Index (PDI), healthy Plant-based Diet Index (hPDI), unhealthy Plant-based Diet Index (uPDI), Healthy Eating Index 2015 (HEI-2015) and EAT-lancet Score] and risk of cholelithiasis. We developed a PRS for cholelithiasis derived from the replication dataset of a prior study (12). The present study also examined the combined associations of dietary patterns and genetic factors with cholelithiasis risk, focusing on the extent to which healthy dietary patterns can mitigate the risk for individuals with high-genetic susceptibility. By exploring these associations, our study may provide valuable insights into optimal dietary recommendations tailored to individuals at genetic risk of cholelithiasis. This study also carries significant implications for public health policies aimed at improving dietary patterns to reduce the prevalence of cholelithiasis in the general population.

## 2 Materials and methods

### 2.1 Study population

The UK biobank was approved by the North West Multi-center Research Ethics Committee (REC ID: 16/NW/0274). This large-scale study has recruited over 500,000 adults, aged between 40 and 69 years, from the UK since 2006 (13–15). Detailed information can be found on the website of the UK Biobank.^1^ Our study focused on participants who had both diet questionnaire and genetic data available. We excluded participants with missing data on the 24-h diet (*n* = 291,126), those with implausible values for total energy intake (<500 kcal or >5,000 kcal; *n* = 363) (16), those who subsequently withdrew from the UK Biobank (*n* = 592), and those with a history of cholelithiasis or cholecystectomy (*n* = 7,557). Participants without available genetic data were also excluded (*n* = 47,476). Finally, a total of 155,323 participants were included in the analysis (Supplementary Figure S1).

### 2.2 Ascertainment of outcomes

Participants were followed up through linkage to the Health and Social Care Information Centre (in England and Wales) and the National Health Service Central Register (in Scotland). The study outcomes were the incidences of cholelithiasis, classified based on the International Classification of Diseases (ICD)-10 codes, specifically coded as K80 (17, 18).

### 2.3 Assessment of dietary patterns

In this study, we analyzed seven dietary patterns: MED, aMED, PDI, hPDI,uPDI, HEI-2015 and EAT-Lancet Score. These patterns were selected based on their established validation scoring systems and their relevance to our study population and research objectives. Previous studies have consistently linked these dietary patterns to various health outcomes, including cardiovascular disease, diabetes, and other metabolic conditions, particularly in Western populations (7, 8). By offering a comprehensive overview of different healthy eating approaches, these patterns allowed us to explore their potential impact on the risk of cholelithiasis.

To accurately assess adherence to these dietary patterns, dietary data were collected through the 24-h dietary recall (Oxford WebQ) which was circulated on four occasions (19). These repeated assessments helped mitigate recall and reporting biases, while also providing a more accurate estimation of participants’ long-term dietary patterns. The quantities of each food and beverage consumed were calculated by multiplying the assigned portion size by the amount reported (20). Energy and nutrient intakes were then estimated based on these quantities and the nutritional composition of each item (21). Following methods described in the previous studies, we constructed dietary pattern scores (5–10). Detailed components and criteria for scoring each dietary pattern are summarized in detail in Supplementary Tables S1–S7. The intake of each component in these dietary patterns was given positive or reverse scores. Higher scores mean greater adherence to healthy eating patterns.

An assessment scale indicating the degree of adherence to the traditional MED was constructed by Trichopoulou et al. (5, 22). The original score was based on the intake of nine items: vegetables, legumes, fruit and nuts, cereals, fish, the ratio of monounsaturated to saturated fat, meat and meat products, dairy and dairy products and alcohol intake. A value of 0 or 1 was assigned to each of the components with the median as the cutoff except alcohol. For beneficial components Intakes above the median of the study subjects received 1 point; all other intakes received 0 points. For components presumed to be detrimental, meat and dairy product consumption less than the median received 1 point. For alcohol, a value of 1 was assigned to the people who consumed between 5 and 25 g per day (Supplementary Table S1). The aMED scoring criteria (6) differ from those of the original MED, separating fruits and nuts into two groups, eliminating the dairy group, including whole-grain products only, including only red and processed meats for the meat group, and assigning alcohol intake between 5 and 15 g/d for 1 point (Supplementary Table S2). The total score for both diets ranges from a minimum of 0 to a maximum of 9.

The PDI was developed by Satija et al. (7) and adapted by Heianza et al. (8) for our FFQ. Briefly, the 18 food groups were ranked into quintiles, and each quintile was assigned a score between 1 and 5, For PDI, participants received a score of 5 for each plant food group for which they were above the highest quintile of consumption, with a score of 1 for consumption below the lowest quintile. Conversely, participants received a reverse score for each animal food group. The PDI was calculated based on the score of each food group (Supplementary Table S3). For hPDI, positive scores were given to healthy plant food groups, and reverse scores to less healthy plant food groups and animal food groups (Supplementary Table S4). Finally, for uPDI, positive scores were given to less healthy plant food groups, and reverse scores to healthy plant food groups and animal food groups (Supplementary Table S5). The total score for all three diets ranged from 18 to 90, with higher scores reflecting greater adherence.

Calculation of the HEI was based on criteria set in the Update of the Healthy Eating Index: HEI-2015 (9), In short, the HEI-2015 consisted of 13 components. Scores assigned to each component ranged from 0 to10, depending on the level of intake. Maximum and minimum scoring standards for the HEI-2015 are shown in Supplementary materials (Supplementary Table S6), For all components, the intakes between the maximum and minimum standards are scored proportionately, with a total score of 100.

The construction of the EAT-Lancet diet score was created by Knuppel et al. (10). This dietary score was based on the 14 key recommended food groups. A value of 0 or 1 was assigned to each component, resulting in possible scores of 0–14 (Supplementary Table S7).

### 2.4 Construction of PRS

In our previous study (12), we used genomic data from the Finnish study to identify 13 SNPs in the population of European ancestry. To evaluate the genetic predisposition of cholelithiasis, these SNPs were utilized to construct a PRS in the current analysis. Detailed information about the SNPs is provided in Supplementary materials (Supplementary Table S8). Briefly, the PRS was calculated by multiplying the number of alleles (0,1,2) for each individual by the effect weight of the corresponding SNPs, and then summing the results to derive PRS for all individuals in the UK Biobank. The formula is as follows:


$$PRS=\overset{n}{\underset{i=1}{\sum }}\beta_{i}\times G_{i}$$

where $\beta_{i}$ represents the effect size of the i -th SNP, and $G_{i}$ is the genotype value for the i -th SNP, indicating the number of risk alleles (0, 1, or 2) present in the individual.

The calculated PRS was stratified into low (quintile 1), intermediate (quintile 2–4), and high risk (quintile 5) for further analysis.

### 2.5 Covariates

At enrollment, information was collected through a touchscreen questionnaire and face-to-face interviews, including socio-demographics factors (age, gender, ethnicity, educational level, overall health status, and longstanding illness), anthropometric measurements (height and weight), lifestyle factors (physical activity, smoking status and alcohol consumption), medical history (multivitamins and mineral supplements). The Index of Multiple Deprivation (IMD) for various research centers, a composite measure derived from national census data on income, employment, education, health, crime, housing, and living environment, was obtained directly from the UK Biobank. Physical activity was assessed using the validated short International Physical Activity Questionnaire (IPAQ) (23), which measures time spent walking, as well as moderate and vigorous activities. Body Mass Index (BMI) was calculated as weight divided by the square of height (kg/m^2^).

### 2.6 Statistical analysis

The person-years of follow-up were calculated from the assessment date for each participant until the endpoint. For the statistical analysis of participant characteristics, continuous variables are described as the mean and SD, and categorical variables are described as numbers and percentages. The Cox proportional hazards regression model was used to assess the association of dietary patterns and PRS with cholelithiasis incidence by calculating hazard ratios (HR) and 95% confidence intervals (CI). The assumption of proportional hazards was confirmed using Schoenfeld residual methods in the UK Biobank and no violation of this assumption was found. To adjust for potential confounders, three types of models were built and added in a stepwise manner. Model 1 was calculated using the Cox model stratified by age, sex, UK Biobank assessment centers, and additionally adjusted for overall health rating, long-standing illness, and education. Model 2 was further adjusted for the index of multiple deprivation, multivitamin use, and mineral use. In model 3, we further adjusted for smoking status, alcohol consumption, physical activity, and body mass index. Kaplan–Meier analysis with log-rank testing was performed to show the cumulative incidence rate across the different PRS categories in all participants. Additionally, we examined the potential nonlinear relationship between dietary patterns and cholelithiasis risk with restricted cubic splines.

In subgroup analyses, the models were reanalyzed by age, gender, educational level, BMI, alcohol consumption, smoking status, and physical activity. To explore whether the associations of dietary patterns with cholelithiasis differed according to genetic risk, we cross-classified participants according to categories of dietary patterns (quartiles 1–4) and PRS (high, intermediate, and low) and conducted joint analyses, using high genetic risk and low adherence to dietary patterns as the reference group. We also conducted several sensitivity analyses to test the robustness of our findings. First, we excluded cholelithiasis cases that occurred within the first 2 or 4 years of follow-up to mitigate any potential reverse causation effects. Second, considering the association between dietary intake and BMI, which can influence health outcomes (24), we did not adjust for BMI to compare the potential over-adjustment bias. All statistical analyses were performed using R software (version 4.3.2) and Python (version 3.7.13), and a two-sided *p*-value < 0.05 was considered statistically significant.

## 3 Results

During a median follow-up of 13.3 years, our study included 155,323 participants (mean age 56.40 years old, 46.70% males). A total of 5,056 cholelithiasis cases were diagnosed. Participants with cholelithiasis were composed of more females (60.80%), poor overall health (6.30%), longstanding illness (40.60%), previous smoking status (39.30%), and low physical activity (18.80%) than participants without cholelithiasis (Table 1). We also compared the baseline characteristics of participants according to the quartiles of dietary score. Participants with higher MED scores were more likely to be older (mean age 56.50 years old), female (57.00%), lower index of multiple deprivation (mean index 14.50), higher-educated (45.80% with college or university degree), less long-standing illness (28.10%), never smokers (57.80%) and high physical activity (34.90%). The other healthy dietary pattern scores had similar characteristics. Conversely, the uPDI exhibited an opposite trend. The detailed characteristics are presented in Supplementary Tables S9-S15.

**Table 1:** Baseline characteristics of participants.

Characteristics	Non-cholelithiasis (*N* = 150,267)	Cholelithiasis (*N* = 5,056)	All participants (*N* = 155,323)
Mean (SD) Age, years	56.30 (7.81)	57.70 (7.68)	56.40 (7.81)
Gender, *N* (%)			
Female	79,754 (53.10)	3,073 (60.80)	82,827 (53.30)
Male	70,513 (46.90)	1,983 (39.20)	72,496 (46.70)
Mean (SD) Index of multiple deprivation	14.70 (11.90)	16.70 (13.50)	14.80 (12.00)
Education level, *N* (%)			
A levels/AS levels or equivalent	19,948 (13.30)	627 (12.40)	20,575 (13.20)
College or University degree	62,666 (41.70)	1,585 (31.30)	64,251 (41.40)
CSEs or equivalent	6,425 (4.30)	269 (5.30)	6,694 (4.30)
NVQ or HND or HNC or equivalent	8,371 (5.60)	331 (6.50)	8,702 (5.60)
O levels/GCSEs or equivalent	32,146 (21.40)	1,219 (24.10)	33,365 (21.50)
Health rate, *N* (%)			
Excellent	30,684 (20.40)	594 (11.70)	31,278 (20.10)
Good	90,334 (60.10)	2,801 (55.40)	93,135 (60.00)
Poor	4,125 (2.70)	321 (6.30)	4,446 (2.90)
Long-standing illness, *N* (%)	42,816 (28.50)	2,051 (40.60)	44,867 (28.90)
Multivitamin use, *N* (%)	22,096 (14.70)	748 (14.80)	22,844 (14.70)
Intake of mineral supplements, *N* (%)	33,013 (22.00)	1,124 (22.20)	34,137 (22.00)
Mean (SD) body mass index, kg/m^2^	26.80 (4.50)	29.30 (5.33)	26.90 (4.56)
Smoking status, *N* (%)			
Current	11,191 (7.40)	383 (7.60)	11,574 (7.50)
Never	85,442 (56.90)	2,672 (52.80)	88,114 (56.70)
Previous	53,333 (35.50)	1,985 (39.30)	55,318 (35.60)
Alcohol consumption, *N* (%)			
Daily or almost daily	36,647 (24.40)	890 (17.60)	37,537 (24.20)
Once or twice a week	37,699 (25.10)	1,309 (25.90)	39,008 (25.10)
One to three times a month	15,705 (10.50)	702 (13.90)	16,407 (10.60)
Special occasions only	12,847 (8.50)	707 (14.00)	13,554 (8.70)
Three or four times a week	39,563 (26.30)	1,041 (20.60)	40,604 (26.10)
Never	7,766 (5.20)	403 (8.00)	8,169 (5.30)
Physical activity, *N* (%)			
High	49,760 (33.10)	1,364 (27.00)	51,124 (32.90)
Low	22,012 (14.60)	950 (18.80)	22,962 (14.80)
Moderate	52,738 (35.10)	1,674 (33.10)	54,412 (35.00)

**Figure 1:**
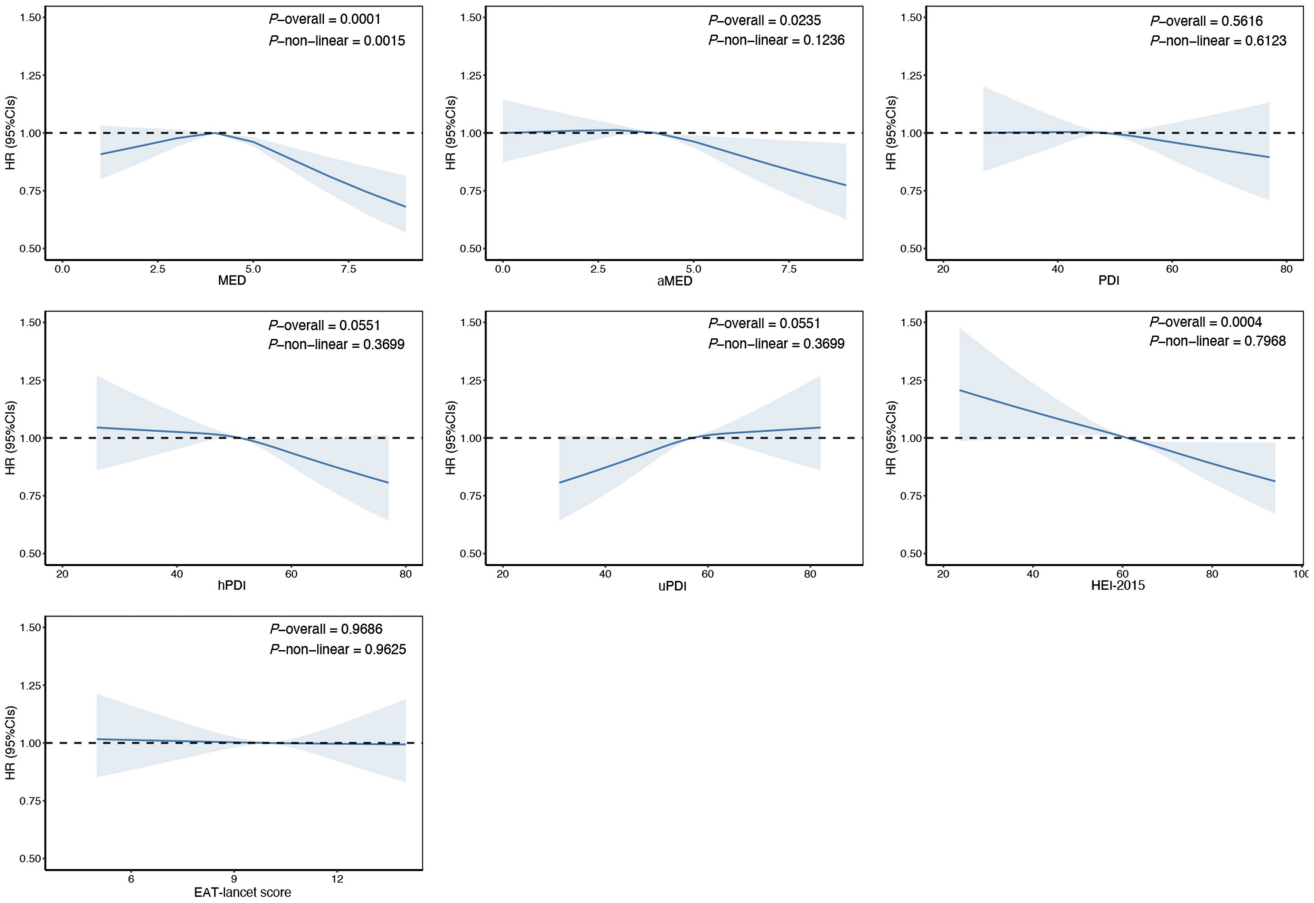
Estimated nonlinear association between dietary patterns and risk of cholelithiasis. Estimated effects were calculated using Cox model stratified by age, sex, UK Biobank assessment centers, and additionally adjusted for overall health rating, long-standing illness, education, index of multiple deprivation, multivitamin use, mineral use, smoking status, alcohol consumption, physical activity and body mass index. This figure shows illustrates the result of using Restricted Cubic Splines (RCS) to analyze the relationship between dietary patterns and cholelithiasis. The reference levels of dietary patterns in these plots (with HR fixed as 1.0) were established as follows: 4 points for Mediterranean Diet Score (MED), 4 points for alternate Mediterranean Diet Score (aMED), 48 points for Plant-based Diet Index (PDI), 51 points for healthy Plant-based Diet Index (hPDI), 57 points for unhealthy Plant-based Diet Index (uPDI), 61 score for Healthy Eating Index 2015 (HEI-2015), 10 points EAT-lancet Score.

The results showed that higher scores on the aMED (HR Q4 compared with Q1: 0.90; 95% CI: 0.83–0.98) and the HEI-2015 (HR Q4 compared with Q1:0.89; 95% CI: 0.82–0.96) were associated with a reduced risk of cholelithiasis. Conversely, higher scores on the uPDI were associated with an increased risk of cholelithiasis in models 1 and 2, however, after adjusting for the full model, the association was no longer statistically significant. Other dietary patterns, such as MED, PDI, hPDI and EAT-lancet scores, showed no significant associations in the fully adjusted models (Table 2). For each SD increase in aMED and HEI-2015 scores, the risk of cholelithiasis decreased by 3% (95% CI: 0.94–0.99) and 6% (95% CI: 0.92–0.97), respectively, with no evidence against linearity (Figure 1). Dietary pattern-associated risk of cholelithiasis did not differ by age, sex, educational level, alcohol consumption, smoking status and physical activity (Supplementary Tables S16-S22).

**Table 2:** The association of dietary patterns with the risk of cholelithiasis.

Dietary patterns	Categories	Cases	Person-years	Model 1		Model 2		Model 3	
				HR (95% CI)	*p* value	HR (95% CI)	*p* value	HR (95% CI)	*p* value
MED									
	Q1	457	159,088.2	1.00 (ref)		1.00 (ref)		1.00 (ref)	
	Q2	1,013	323,369.7	1.07 (0.96–1.19)	0.248	1.08 (0.97–1.21)	0.176	1.07 (0.96–1.20)	0.218
	Q3	1,296	433,380.8	1.03 (0.93–1.15)	0.551	1.05 (0.94–1.16)	0.426	1.04 (0.94–1.16)	0.451
	Q4	2,290	858,317.9	0.96 (0.87–1.06)	0.428	0.97 (0.87–1.07)	0.541	0.99 (0.89–1.09)	0.815
	Per SD increase			0.95 (0.92–0.97)	<0.001	0.95 (0.92–0.97)	<0.001	0.96 (0.93–0.99)	0.004
aMED									
	Q1	1,260	402,074.2	1.00 (ref)		1.00 (ref)		1.00 (ref)	
	Q2	1,246	407,551.9	1.01 (0.93–1.09)	0.787	1.01 (0.93–1.09)	0.853	1.02 (0.94–1.11)	0.585
	Q3	1,176	404,051.9	0.99 (0.91–1.07)	0.738	0.99 (0.91–1.07)	0.802	1.01 (0.94–1.10)	0.723
	Q4	1,374	560,478.7	0.86 (0.80–0.93)	<0.001	0.86 (0.80–0.93)	<0.001	0.90 (0.83–0.98)	0.010
	Per SD increase			0.95 (0.92–0.97)	<0.001	0.95 (0.92–0.98)	<0.001	0.97 (0.94–0.99)	0.018
PDI									
	Q1	1,271	415,341.5	1.00 (ref)		1.00 (ref)		1.00 (ref)	
	Q2	1,128	383,833.2	1.00 (0.92–1.08)	0.933	1.00 (0.92–1.08)	0.922	1.02 (0.94–1.10)	0.702
	Q3	1,297	467,705.4	0.97 (0.89–1.04)	0.378	0.97 (0.89–1.05)	0.395	0.99 (0.92–1.07)	0.847
	Q4	1,360	507,276.5	0.97 (0.89–1.04)	0.383	0.97 (0.89–1.04)	0.387	1.01 (0.94–1.10)	0.767
	Per SD increase			0.97 (0.94–1.00)	0.028	0.97 (0.94–1.00)	0.028	0.99 (0.96–1.02)	0.368
hPDI									
	Q1	1,134	379,507.9	1.00 (ref)		1.00 (ref)		1.00 (ref)	
	Q2	1,331	452,941.2	0.96 (0.88–1.03)	0.266	0.96 (0.89–1.04)	0.351	1.01 (0.93–1.09)	0.872
	Q3	1,374	489,072.9	0.89 (0.83–0.97)	0.006	0.9 (0.83–0.97)	0.008	0.97 (0.89–1.05)	0.422
	Q4	1,217	452,634.7	0.85 (0.79–0.93)	<0.001	0.86 (0.79–0.94)	<0.001	0.96 (0.88–1.04)	0.331
	Per SD increase			0.93 (0.90–0.95)	<0.001	0.93 (0.90–0.96)	0.298	0.97 (0.94–1.00)	0.029
uPDI									
	Q1	973	372,243.3	1.00 (ref)		1.00 (ref)		1.00 (ref)	
	Q2	1,317	466,473.3	1.08 (1.00–1.18)	0.062	1.08 (0.99–1.18)	0.071	1.06 (0.97–1.15)	0.206
	Q3	1,405	479,634.6	1.14 (1.05–1.23)	0.002	1.13 (1.04–1.23)	0.003	1.07 (0.98–1.16)	0.130
	Q4	1,361	455,805.5	1.20 (1.10–1.30)	<0.001	1.19 (1.09–1.29)	<0.001	1.08 (0.99–1.17)	0.085
	Per SD increase			1.08 (1.05–1.11)	<0.001	1.08 (1.05–1.11)	<0.001	1.03 (1.00–1.06)	0.029
HEI-2015									
	Q1	1,343	444,174.1	1.00 (ref)		1.00 (ref)		1.00 (ref)	
	Q2	1,338	443,381.3	1.02 (0.94–1.10)	0.691	1.03 (0.95–1.11)	0.471	1.03 (0.96–1.12)	0.419
	Q3	1,228	443,827.7	0.94 (0.87–1.01)	0.100	0.94 (0.87–1.02)	0.152	0.95 (0.88–1.03)	0.195
	Q4	1,147	442,773.6	0.87 (0.80–0.94)	0.001	0.87 (0.81–0.95)	0.001	0.89 (0.82–0.96)	0.005
	Per SD increase			0.94 (0.91–0.96)	<0.001	0.94 (0.91–0.97)	<0.001	0.94 (0.92–0.97)	<0.001
EAT-lancet score									
	Q1	943	314,850.1	1.00 (ref)		1.00 (ref)		1.00 (ref)	
	Q2	1,192	415,361.4	0.95 (0.87–1.04)	0.258	0.95 (0.87–1.03)	0.226	0.97 (0.89–1.06)	0.457
	Q3	1,419	494,105.1	0.96 (0.89–1.04)	0.353	0.96 (0.89–1.05)	0.380	0.99 (0.91–1.08)	0.906
	Q4	1,502	549,840.1	0.93 (0.86–1.01)	0.086	0.94 (0.86–1.02)	0.117	1.00 (0.92–1.08)	0.917
	Per SD increase			0.97 (0.94–1.00)	0.038	0.97 (0.95–1.00)	0.069	1.00 (0.97–1.03)	0.896

HR, hazard ratio; CI, confidence interval; SD, standard deviation; Q, quantile; AMED, Alternate Mediterranean Diet; HEI-2015, Healthy Eating Index 2015; HPDI, Healthful Plant-based Diet Index. Model 1: Estimated effects were calculated using Cox model stratified by age, sex, UK Biobank assessment centers, and additionally adjusted for overall health rating, long-standing illness and education. Model 2: additionally adjusted for index of multiple deprivation, multivitamin use and mineral use. Model 3: additionally adjusted for smoking status, alcohol consumption, physical activity and body mass index.

**Figure 2:**
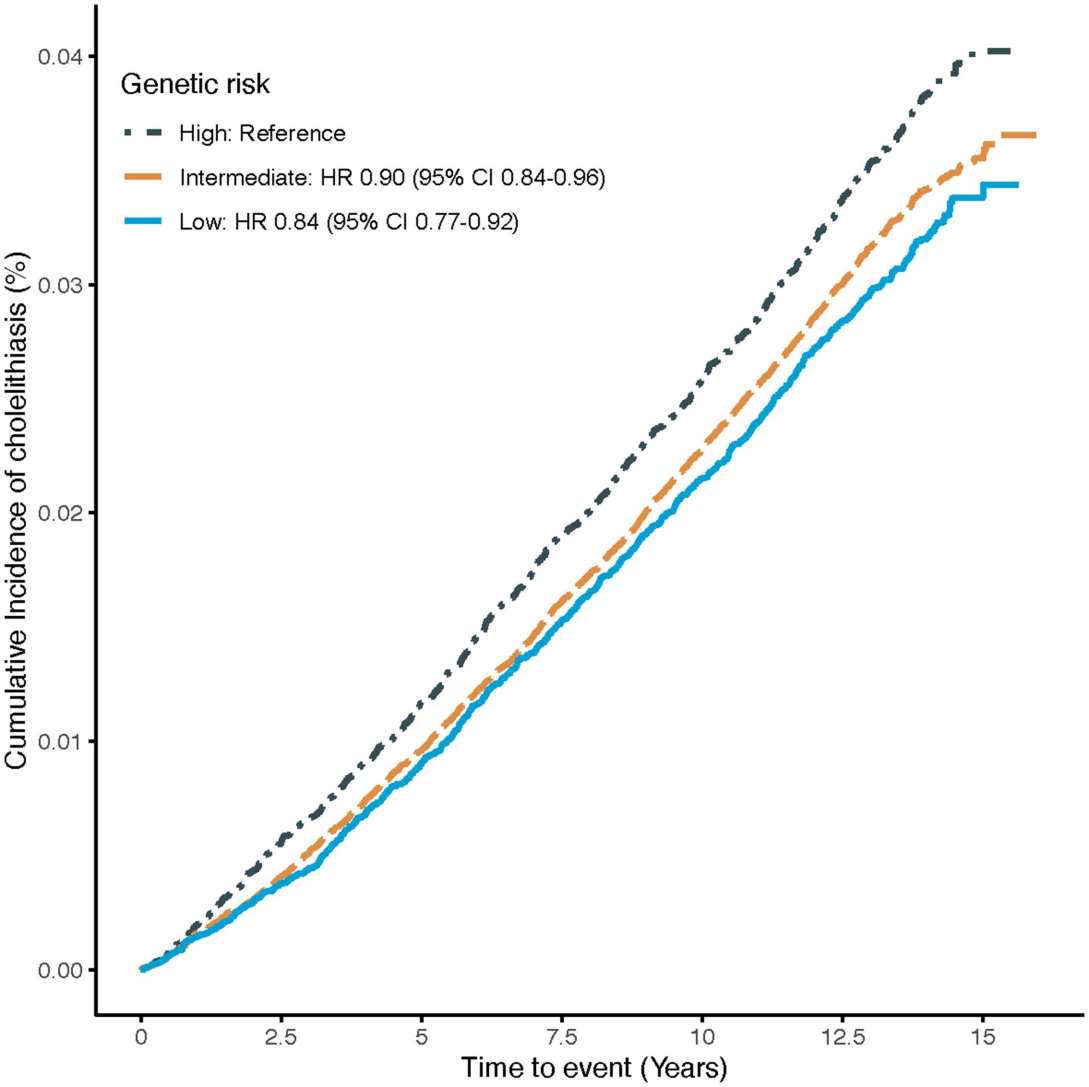
Cumulative cholelithiasis incidences for high, intermediate and low-genetic risk groups. Estimated effects were calculated using Cox model stratified by age, sex, UK Biobank assessment centers, and additionally adjusted for overall health rating, long-standing illness, education, index of multiple deprivation, multivitamin use, mineral use, smoking status, alcohol consumption, physical activity and body mass index. The figure shows the cumulative incidence of cholelithiasis in low (quintile 1), intermediate (quintiles 2–4), and high (quintile 5) genetic risk groups. HR, Hazard ratio; CI, confidence interval.

The association of genetic risk with the risk of cholelithiasis is shown in Figure 2. Compared to participants with high genetic risk, those with intermediate or low genetic risk had a 10% (95%CI: 0.84–0.96) and 16% (95%CI: 0.77–0.92) lower risk of cholelithiasis, respectively, suggesting that the PRS could effectively stratify risk for idividuals with cholelithiasis.

**Figure 3:**
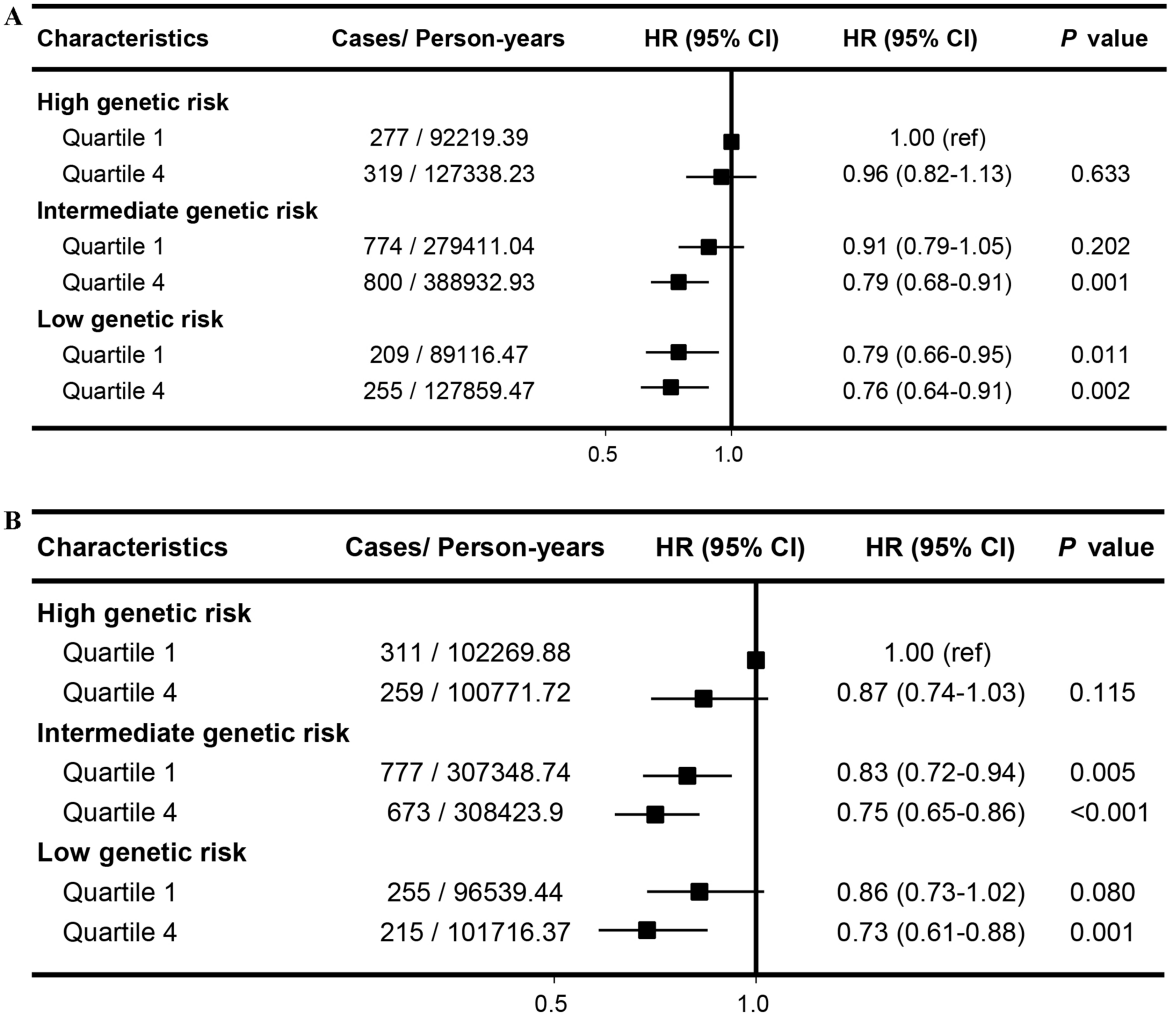
Joint effects and interactions between aMED **(A)**, HEI-2015 **(B)** and PRS on cholelithiasis risk. Estimated effects were calculated using Cox model stratified by age, sex, UK Biobank assessment centers, and additionally adjusted for overall health rating, long-standing illness, education, index of multiple deprivation, multivitamin use, mineral use, smoking status, alcohol consumption, physical activity and body mass index. ref., reference; HR, Hazard ratio; CI, confidence interval.

Our findings indicated that there was a combined association of aMED, HEI-2015 and genetic predisposition with the risk of cholelithiasis in a dose–response relationship. Compared with participants at high genetic risk and low aMED score, those at low genetic risk and high aMED scores showed the lowest risk of cholelithiasis (HR: 0.76; 95% CI: 0.64–0.91; Figure 3A). The combined impact of low genetic risk and high HEI-2015 significantly reduced cholelithiasis risk, with an HR of 0.73 (95% CI: 0.61–0.88; Figure 3B). This association remained unchanged after lagging the exposure for 2 or 4 years, and remained stable when not adjusting for BMI categories (Supplementary Figures S23–29).

## 4 Discussion

In this large-scale cohort study, we examined the associations between various dietary patterns and the risk of cholelithiasis, as well as the combined associations of dietary patterns and genetic predisposition with cholelithiasis risk. Our results indicated that higher adherence to the aMED and HEI-2015 were associated with a reduced risk of cholelithiasis. These associations remained consistent across subgroup and sensitivity analyses. Other dietary patterns, such as the MED, PDI, hPDI, uDPI, and EAT-lancet scores, showed no significant associations in the fully adjusted models. We also generated a composite PRS to evaluate the genetic risk of cholelithiasis and found that participants with low genetic risk had a 16% lower risk of cholelithiasis compared to those with high genetic risk. Furthermore, the joint analysis revealed that individuals with low genetic risk and high adherence to either aMED or HEI-2015 diets exhibited a lower cholelithiasis risk compared to those with high genetic risk and low adherence to these dietary patterns. Overall, the results suggested that the risk of cholelithiasis attributable to an individual’s genetic predisposition could be offset by adherence to a healthy dietary pattern.

Previous studies have primarily focused on the intake of a single food group or isolated nutrients in relation to cholelithiasis (25–28). However, understanding the impact of dietary patterns rather than individual nutrients or specific foods offers a more comprehensive perspective by considering the combined and interactive influences of various dietary components (29–31). Our findings underscore the significant reduction in cholelithiasis risk associated with high adherence to the aMED and HEI-2015 dietary patterns. Both the aMED and HEI-2015 patterns advocate for diets rich in fruits, vegetables, whole grains, and healthy fats. They emphasize the consumption of a diverse range of vegetables and fruits, prioritize healthy fats such as those found in olive oil, nuts, and seeds, and recommend whole grains over refined grains. Additionally, these patterns promote high-quality protein sources such as fish, seafood, lean meats, legumes, and nuts, while limiting intake of added sugars, salt, red meat, and processed foods. These dietary components are likely to confer anti-inflammatory and antioxidant benefits, which may inhibit the formation of cholelithiasis (32, 33). Conversely, the increased risk associated with uPDI highlights the detrimental impact of unhealthy dietary patterns characterized by high consumption of refined grains, sugars, and unhealthy fats. These dietary habits are known to promote obesity and dyslipidemia, key risk factors for gallstone formation (34–36). The non-significant results observed for the MED, PDI, hPDI, and EAT-Lancet scores may be attributed to variations in dietary adherence across different patterns and potential residual confounding factors. This suggests a need for further investigation into specific components of these dietary patterns and their interactions with other lifestyle factors.

Several studies have investigated the relationship between dietary patterns and the risk of cholelithiasis, reporting varying findings across different populations. For instance, a small case–control study of 305 participants demonstrated that adherence to a healthy dietary pattern characterized by a high intake of vegetables, fruits, low-fat dairy products, whole grains, nuts, legumes, and spices was significantly associated with a lower prevalence of cholelithiasis. Conversely, unhealthy dietary patterns showed the opposite association (37). In cohort studies, diverse dietary patterns have been assessed for their impact on cholelithiasis risk. For example, a prospective analysis within the French E3N cohort found that higher adherence to the Mediterranean diet (MED) was linked to a reduced risk of cholecystectomy (38). Another cohort study indicated that a plant-based diet was associated with a lower incidence of symptomatic gallstone disease in women, but not in men, residing in Taiwan (39). Similarly, a comparative study among US male health professionals revealed that adherence to three healthy diet patterns—the DASH, aMED, and HEI-2010—was associated with approximately a 35% lower risk of symptomatic gallstone disease (40). Conversely, a cross-sectional analysis from the China Multi-Ethnic Cohort Study reported contradictory findings, where a rice-based dietary pattern was associated with a lower risk of cholelithiasis, while aMED showed an opposite association (41). Despite these studies, there remains inconsistency in the impact of dietary patterns on cholelithiasis risk across different populations. Our study adds new insights to this field by contributing findings that further clarify the relationship between dietary patterns and cholelithiasis risk. With the development of genomics and biobanks, PRS have emerged as valuable tools for evaluating genetic risk and identifying individuals at increased risk for various diseases, thereby aiding in prevention strategies (8, 12, 42, 43). In this study, we further explored the role of genetic risk in cholelithiasis by quantifying and stratifying it using a PRS and examining its interaction with dietary patterns. We observed a linear dose–response association between the 13-SNP PRS and cholelithiasis risk, consistent with trends seen in previous studies on cholelithiasis (11, 12). Inherited susceptibility plays a significant role in the development of several diseases, including cholelithiasis, and warrants close attention (8, 11, 12, 43). Our findings suggest that understanding genetic risk factors for cholelithiasis can enhance clinical and personal decision-making processes regarding risk management. This insight could lead to more targeted prevention strategies and improved patient outcomes in clinical practice.

There are several potential biological mechanisms that could explain how healthy dietary patterns, such as aMED and HEI-2015, influence the risk of cholelithiasis. First, specific components within these dietary patterns may mitigate the risk of gallstone formation by regulating bile acid metabolism, maintaining cholesterol homeostasis, and modulating inflammatory processes. For example, the aMED and HEI-2015 diets are rich in dietary fiber, which may reduce cholelithiasis risk by enhancing the production of short-chain fatty acids, lowering cholesterol saturation in bile, and improving insulin resistance (44). Additionally, antioxidant-rich foods, such as fruits and vegetables, may provide further protection by reducing inflammation in the gallbladder (16). From a genetic perspective, variations in genes involved in lipid metabolism or bile acid transport could influence how diet affects cholelithiasis risk. For instance, APOE gene variants, which are closely linked to cholesterol metabolism, may heighten the risk of cholelithiasis by altering an individual’s response to a high-fat diet (45). These biological mechanisms not only support the epidemiological evidence from our population-based cohort study but also underscore the biological plausibility of healthy dietary patterns in reducing cholelithiasis risk. Collectively, these findings highlight the significant role that dietary patterns may play in the prevention of cholelithiasis, particularly in individuals with genetic susceptibility.

Importantly, our study expands existing knowledge by illustrating the interaction between dietary patterns and genetic susceptibility to cholelithiasis (37–41). We found that the excess risks associated with genetic predisposition can be partly offset by adherence to healthy dietary patterns. Individuals with intermediate and low genetic risk who adhered closely to the HEI-2015 dietary pattern had a 25 and 27% lower risk of cholelithiasis, respectively. Participants with both high dietary scores and low genetic risk exhibited the lowest risk, with hazard ratios of 0.76 for aMED and 0.73 for HEI-2015. These findings underscore the necessity of maintaining healthy dietary patterns to prevent cholelithiasis, suggesting that a healthy diet can partly counteract the negative influences of genetic predisposition. The observed synergistic interaction highlights the potential of dietary interventions in reducing cholelithiasis risk across different genetic backgrounds. However, the precise mechanisms underlying these interactions remain uncertain and require further investigation.

To our knowledge, this study is the first large prospective cohort study to investigate the combined associations of diverse dietary patterns and PRS with cholelithiasis. One of the key strengths of our research is the large-scale sample size, which, along with its prospective design, long-term follow-up, and extensive assessment of dietary intake (14), allowed us to accurately explore the combined impacts of genetic risk and dietary patterns on cholelithiasis. We also collected detailed information on multiple confounders and adjusted for them comprehensively, conducting sensitivity analyses that enhance the reliability and generalizability of our findings. However, despite our robust methodology, our study has some limitations. The observational nature of the study limits causal inference, self-reported dietary data may introduce recall and reporting biases and a single dietary assessment may have limited representation of long-term dietary habits. Furthermore, although we adjusted for a wide range of confounders, the possibility of residual confounding and measurement errors cannot be entirely ruled out. In particular, unmeasured factors such as physical activity levels, gut microbiota composition, and detailed socioeconomic variables may influence both dietary patterns and the risk of cholelithiasis. Ignoring these factors could lead to residual confounding, potentially affecting the robustness of our findings. Additionally, while the PRS is a powerful tool for analyzing the interaction between genetic risk and environmental factors, it has limitations. The PRS was built using SNPs associated with cholelithiasis identified in individuals of European ancestry, and our study focused on participants from the UK. This may limit its applicability to other ethnic groups, as genetic architectures can differ significantly. Moreover, the PRS does not consider environmental and lifestyle factors, which may reduce its predictive accuracy. Finally, we acknowledge that other dietary patterns, including traditional dietary patterns from different cultures, could also be relevant to understanding the risk of cholelithiasis. However, due to specific limitations in our dataset and the focused scope of our study, we did not include these patterns (38, 40, 41, 46). This exclusion may limit the generalizability of our findings to broader populations. Future research should explore additional dietary patterns to provide a more comprehensive understanding of the dietary influences on cholelithiasis risk, validate these scores in non-European populations, integrate other biomarkers, or include environmental factors to enhance PRS accuracy and relevance. Additionally, randomized controlled trials and objective dietary assessments would be beneficial to validate these findings.

## 5 Conclusion

This prospective study indicated that higher aMED or HEI-2015 scores and lower PRS were independently and jointly associated with a reduced risk of cholelithiasis. Adherence to these two healthy dietary patterns may partially offset the genetic predisposition to cholelithiasis. These findings highlight the potential of dietary interventions in reducing the risk of cholelithiasis and provide evidence supporting the development of preventive health strategies.

## Data Availability

Publicly available datasets were analyzed in this study. This data can be found here: https://www.ukbiobank.ac.uk/.

## Footnotes

^1^ https://www.ukbiobank.ac.uk/
