# Supplementary material for: Dietary patterns, genetic predisposition, and risk of cholelithiasis: a large-scale prospective cohort study
Source: Front Nutr. 2024 Sep 27;11:1469789. doi: 10.3389/fnut.2024.1469789 (PMC11466834; doi:10.3389/fnut.2024.1469789)
Supplement: Supplementary file 1 [file Data_Sheet_1.pdf]

Supplementary Material

Table of contents

Supplementary Table S1. Components and scoring criteria of the Mediterranean Diet (MED) score. 3

Supplementary Table S2. Components and scoring criteria of the alternative Mediterranean Diet (aMED) score. 4

Supplementary Table S3. Components and scoring criteria of the overall Plant-based Diet Index (PDI). 5

Supplementary Table S4. Components and scoring criteria of the healthy Plant-based Diet Index (hPDI). 7

Supplemental Table S5. Components and scoring criteria of the unhealthy Plant-based Diet Index (uPDI). 9

Supplementary Table S6. Components and scoring criteria of the Healthy Eating Index 2015 (HEI-2015). 11

Supplementary Table S7. Components and scoring criteria of EAT-Lancet Score. 13

Supplementary Table S8. Single-nucleotide polymorphisms used to construct the genetic risk score for cholelithiasis 14

Supplementary Table S9. Baseline characteristics of participants, according to the quartiles of MED. 15

Supplementary Table S10. Baseline characteristics of participants, according to the quartiles of aMED. 17

Supplementary Table S11. Baseline characteristics of participants, according to the quartiles of PDI. 19

Supplementary Table S12. Baseline characteristics of participants, according to the quartiles of hPDI. 21

Supplementary Table S13. Baseline characteristics of participants, according to the quartiles of uPDI. 23

Supplementary Table S14. Baseline characteristics of participants, according to the quartiles of HEI-2015 25

Supplementary Table S15. Baseline characteristics of participants, according to the quartiles of EAT-lancet score. 27

Supplementary Table S16. Subgroup analysis for the association between MED and risk of cholelithiasis incidence. 29

Supplementary Table S17. Subgroup analysis for the association between aMED and risk of cholelithiasis incidence. 30

Supplementary Table S18. Subgroup analysis for the association between PDI and risk of cholelithiasis incidence. 31

Supplementary Table S19. Subgroup analysis for the association between hPDI and risk of cholelithiasis incidence. 32

Supplementary Table S20. Subgroup analysis for the association between uPDI and risk of cholelithiasis incidence. ***33***

Supplementary Table S21. Subgroup analysis for the association between HEI-2015 and risk of cholelithiasis incidence. ***34***

Supplementary Table S22. Subgroup analysis for the association between EAT-lancet score and risk of cholelithiasis incidence. ***35***

Supplementary Table S23. Sensitivity analyses of the cholelithiasis risk according to MED and the level of genetic risk. ***36***

Supplementary Table S24. Sensitivity analyses of the cholelithiasis risk according to aMED and the level of genetic risk. ***37***

Supplementary Table S25. Sensitivity analyses of the cholelithiasis risk according to PDI and the level of genetic risk. ***38***

Supplementary Table S26. Sensitivity analyses of the cholelithiasis risk according to hPDI and the level of genetic risk. ***39***

Supplementary Table S27. Sensitivity analyses of the cholelithiasis risk according to uPDI and the level of genetic risk. ***40***

Supplementary Table S28. Sensitivity analyses of the cholelithiasis risk according to HEI-2015 and the level of genetic risk. ***41***

Supplementary Table S29. Sensitivity analyses of the cholelithiasis risk according to EAT-lancet score and the level of genetic risk. ***42***

Supplementary Figure S1. Flow diagram of participants in the present study. ***43***

Supplementary Figure S2. Joint effects and interactions of dietary patterns and PRS on cholelithiasis risk. ***44***

Supplementary Table S1. Components and scoring criteria of the Mediterranean Diet (MED) score.

Components	Food	Score of 1	Score of 0
1.vegetables	coleslaw, side salad, avocado, beetroot, broccoli, butternut squash, cabbage/kale, carrot, cauliflower, celery, courgette, cucumber, garlic, leek, lettuce, mushroom, onion, parsnip, sweet pepper, spinach, other vegetables	for each component, intakes above the median	for each component, intakes below the median
2.legumes	baked bean, pulses, broad bean, tofu		
3.fruits and nuts	fruit juice, stewed fruit, prune, dried fruit, mixed fruit, apple, banana, berry, cherry, grapefruit, grape, mango, melon, orange, satsuma, other fruit， salted peanuts, unsalted peanuts, salted nuts, unsalted nuts, seeds		
4.cereals	porridge, muesli, oat crunch, sweetened cereal, plain cereal, bran cereal, whole-wheat cereal, other cereal, whole-meal pasta, brown rice, couscous， bread, garlic bread, crispbread, oatcakes, other bread, white pasta, white rice, other grain, pancake, scotch pancake, danish pastry, scone, cereal bar, savoury biscuits, cheesy biscuits		
5.fish	tinned tuna, oily fish, breaded fish, battered fish, white fish, prawns, lobster/crab, shellfish, other fish		
6. MUFA: SFA ratio	monounsaturated fatty acids, saturated fatty acids		
7.meat and meat product	sausage, beef, pork, lamb, bacon, ham, liver	for each component, intakes below the median	for each component, intakes above the median
8.dairy and dairy product	fat hard cheese, hard cheese, soft cheese, blue cheese, low fat cheese spread, cheese spread, cottage cheese, feta, mozzarella, goats cheese, other cheese		
9.alcohol	alcohol	5 g/d ≤ alcohol ≤25 g/d	alcohol <5g/d or >25 g/d
total score		9	0

Abbreviations: MUFA, monounsaturated fatty acid; SFA, saturated fatty acid.

Supplementary Table S2. Components and scoring criteria of the alternative Mediterranean Diet (aMED) score.

Components	Food	Score of 1	Score of 0
1.vegetables	coleslaw, side salad, avocado, beetroot, broccoli, butternut squash, cabbage/kale, carrot, cauliflower, celery, courgette, cucumber, garlic, leek, lettuce, mushroom, onion, parsnip, sweet pepper, spinach, other vegetables		
2.legumes	baked bean, pulses, broad bean, tofu		
3.fruits	fruit juice, stewed fruit, prune, dried fruit, mixed fruit, apple, banana, berry, cherry, grapefruit, grape, mango, melon, orange, satsuma, other fruit		
4.nuts	salted peanuts, unsalted peanuts, salted nuts, unsalted nuts, seeds	for each component, intakes above the median	for each component, intakes below the median
5.whole grains	muesli, oat crunch, sweetened cereal, plain cereal, bran cereal, whole-wheat cereal, other cereal, oatcakes, whole-meal pasta, brown rice, couscous, cereal bar		
6.fish	tinned tuna, oily fish, breaded fish, battered fish, white fish, prawns, lobster/crab, shellfish, other fish		
7. MUFA: SFA ratio	monounsaturated fatty acids, saturated fatty acids		
8.red and processed meat	sausage, beef, pork, lamb, bacon, ham, liver	for each component, intakes below the median	for each component, intakes above the median
9.alcohol	alcohol	5 g/d ≤ alcohol ≤15 g/d	alcohol <5g/d or >15 g/d
total score		9	0

Abbreviations: MUFA, monounsaturated fatty acid; SFA, saturated fatty acid.

Supplementary Table S3. Components and scoring criteria of the overall Plant-based Diet Index (PDI).

Components	Food	Criteria for maximum score [a]	Criteria for minimum score
1.whole grains	porridge, muesli, oat crunch, sweetened cereal, plain cereal, bran cereal, whole-wheat cereal, other cereal, whole-meal pasta, brown rice, couscous		
2.fruits	avocado, stewed fruit, mixed fruit, apple, banana, berry, cherry, grapefruit, grape, mango, melon, orange, satsuma, peach/nectarine, pear, pineapple, plum, other fruits		
3.vegetables	coleslaw, side salad, beetroot, broccoli, butternut squash, cabbage/kale, carrot,cauliflower, celery, courgette, cucumber, garlic, leek, lettuce, mushroom, onion, parsnip, pea, sweet pepper, spinach, sprouts, sweetcorn, sweet potato, fresh tomato, tinned tomato, turnip/swede, watercress, other vegetables		
4.nuts	salted peanuts, unsalted peanuts, salted nuts, unsalted nuts, seeds		
5.legumes	baked bean, pulses, broad bean, green bean, tofu		
6.vegetable oils	Oil-based salad dressing, vegetable oil used for cooking		
7.tea & coffee	instant coffee, filtered coffee, cappuccino, latte, espresso, other coffee type, decaffeinated coffee, standard tea, rooibos tea, green tea, herbal tea, other tea	for each component, intakes above the highest quintile score 5	for each component, intakes below the lowest quintile score 1
8.fruit juices	squash, orange juice, grapefruit juice, pure fruit/vegetable juice		
9.refined grains	sliced bread, baguette, bap, bread roll, naan bread, garlic bread, crispbread, oatcakes, other bread, white pasta, white rice, other grain, pancake, scotch pancake, danish pastry, scone, cereal bar, savoury biscuits, cheesy biscuits		
10.potatoes	fried potatoes, boiled/baked potatoes, mashed potato, crisp		
11.sugar sweetened. beverages	fizzy drink, fruit smoothie, dairy smoothie,other drink		
12.sweets and desserts	hot chocolate, prune, dried fruit, snackpot, double crust pastry, single crust pastry, crumble, milk-based pudding, fruitcake, cake, doughnut, sponge pudding, cheesecake, other dessert, chocolate bar, white chocolate, milk chocolate, dark chocolate, chocolate-covered raisin, chocolate sweet, diet sweets, sweets, chocolate-covered biscuits, chocolate biscuits, sweet biscuits, other sweets, other savoury snack		

13.animal fat	butter/margarine on bread/crackers, butter/margarine added to potatoes		
14.dairy	milk, flavoured milk, yogurt, ice-cream, low fat hard cheese, hard cheese, soft cheese, blue cheese, low fat cheese spread, cheese spread, cottage cheese, feta, mozzarella, goats cheese, other cheese		
15.egg	whole egg, omelette, eggs in sandwiches, scotch egg, other egg	for each component, intakes below the lowest quintile score 5	for each component, intakes above the highest quintile score 1
16.fish or seafood	tinned tuna, oily fish, breaded fish, battered fish, white fish, prawns, lobster/crab, shellfish, other fish		
17.meat	sausage, beef, pork, lamb, crumbed or deep-fried poultry, poultry, bacon, ham, liver, other meat		
18.miscellaneous animal-based foods	pizza, chowder or cream soup		
total score		90	18

[a] Each component is divided into quintiles of consumption, and each quintile is assigned a score of 5 to 1.

Supplementary Table S4. Components and scoring criteria of the healthy Plant-based Diet Index (hPDI).

Components	Food	Criteria for maximum score [a]	Criteria for minimum score
1.whole grains	porridge, muesli, oat crunch, sweetened cereal, plain cereal, bran cereal, whole-wheat cereal, other cereal, whole-meal pasta, brown rice, couscous	for each component, intakes above the highest quintile score 5	for each component, intakes below the lowest quintile score 1
2.fruits	avocado, stewed fruit, mixed fruit, apple, banana, berry, cherry, grapefruit, grape, mango, melon, orange, satsuma, peach/nectarine, pear, pineapple, plum, other fruit		
3.vegetables	coleslaw, side salad, beetroot, broccoli, butternut squash, cabbage/kale, carrot, cauliflower, celery, courgette, cucumber, garlic, leek, lettuce, mushroom, onion, parsnip, pea, sweet pepper, spinach, sprouts, sweetcorn, sweet potato, fresh tomato, tinned tomato, turnip/swede, watercress, other vegetables		
4.nuts	salted peanuts, unsalted peanuts, salted nuts, unsalted nuts, seeds		
5.legumes	baked bean, pulses, broad bean, green bean, tofu		
6.vegetable oils	Oil-based salad dressing, vegetable oil used for cooking		
7.tea & coffee	instant coffee, filtered coffee, cappuccino, latte, espresso, other coffee type, decaffeinated coffee, standard tea, rooibos tea, green tea, herbal tea, other tea		
8.fruit juices	squash, orange juice, grapefruit juice, pure fruit/vegetable juice	for each component, intakes below the lowest quintile score 5	for each component, intakes above the highest quintile score 1
9.refined grains	sliced bread, baguette, bap, bread roll, naan bread, garlic bread, crispbread, oatcakes, other bread, white pasta, white rice, other grain, pancake, scotch pancake, danish pastry, scone, cereal bar, savoury biscuits, cheesy biscuits		
10.potatoes	fried potatoes, boiled/baked potatoes, mashed potato, crisp		
11.sugar sweetened beverages	fizzy drink, fruit smoothie, dairy smoothie,other drink		
12.sweets and desserts	hot chocolate, prune, dried fruit, snackpot, double crust pastry, single crust pastry, crumble, milk-based pudding, fruitcake, cake, doughnut, sponge pudding, cheesecake, other dessert, chocolate bar, white chocolate, milk chocolate, dark chocolate, chocolate-covered raisin, chocolate sweet, diet sweets, sweets, chocolate-covered biscuits, chocolate biscuits, sweet biscuits, other sweets, other savoury snack		

13.animal fat	butter/margarine on bread/crackers, butter/margarine added to potatoes		
14.dairy	milk, flavoured milk, yogurt, ice-cream, low fat hard cheese, hard cheese, soft cheese, blue cheese, low fat cheese spread, cheese spread,cottage cheese, feta, mozzarella, goats cheese, other cheese		
15.egg	whole egg, omelette, eggs in sandwiches, scotch egg, other egg		
16.fish or seafood	tinned tuna, oily fish, breaded fish, battered fish, white fish, prawns, lobster/crab, shellfish, other fish		
17.meat	sausage, beef, pork, lamb, crumbed or deep-fried poultry, poultry, bacon, ham, liver, other meat		
18. miscellaneous animal-based foods	pizza, chowder or cream soup		
total score		90	18

[a] Each component is divided into quintiles of consumption, and each quintile is assigned a score of 5 to 1.

Supplemental Table S5. Components and scoring criteria of the unhealthy Plant-based Diet Index (uPDI).

Components	Food	Criteria for maximum score [a]	Criteria for minimum score
1.whole grains	porridge, muesli, oat crunch, sweetened cereal, plain cereal, bran cereal, whole-wheat cereal, other cereal, whole-meal pasta, brown rice, couscous	for each component, intakes below the lowest quintile score 5	for each component, intakes below the highest quintile score 1
2.fruits	avocado, stewed fruit, mixed fruit, apple, banana, berry, cherry, grapefruit, grape, mango, melon, orange, satsuma, peach/nectarine, pear, pineapple, plum, other fruit		
3.vegetables	coleslaw, side salad, beetroot, broccoli, butternut squash, cabbage/kale, carrot,cauliflower, celery, courgette, cucumber, garlic, leek, lettuce, mushroom, onion, parsnip, pea, sweet pepper, spinach, sprouts, sweetcorn, sweet potato, fresh tomato, tinned tomato, turnip/swede, watercress, other vegetables		
4.nuts	salted peanuts, unsalted peanuts, salted nuts, unsalted nuts, seeds		
5.legumes	baked bean, pulses, broad bean, green bean, tofu		
6.vegetable oils	Oil-based salad dressing, vegetable oil used for cooking		
7.tea & coffee	instant coffee, filtered coffee, cappuccino, latte, espresso, other coffee type, decaffeinated coffee, standard tea, rooibos tea, green tea, herbal tea, other tea		
8.fruit juices	squash, orange juice, grapefruit juice, pure fruit/vegetable juice	for each component, intakes above the highest quintile score 5	for each component, intakes below the lowest quintile score 1
9.refined grains	sliced bread, baguette, bap, bread roll, naan bread, garlic bread, crispbread, oatcakes, other bread, white pasta, white rice, other grain, pancake, scotch pancake, danish pastry, scone, cereal bar, savoury biscuits, cheesy biscuits		
10.potatoes	fried potatoes, boiled/baked potatoes, mashed potato, crisp		
11.sugar sweetened beverages	fizzy drink, fruit smoothie, dairy smoothie,other drink		
12.sweets and desserts	hot chocolate, prune, dried fruit, snackpot, double crust pastry, single crust pastry, crumble, milk-based pudding, fruitcake, cake, doughnut, sponge pudding, cheesecake, other dessert, chocolate bar, white chocolate, milk chocolate, dark chocolate, chocolate-covered raisin, chocolate sweet, diet sweets, sweets, chocolate-covered biscuits, chocolate biscuits, sweet biscuits, other sweets, other savoury snack		

13.animal fat	butter/margarine on bread/crackers, butter/margarine added to potatoes		
14.dairy	milk, flavoured milk, yogurt, ice-cream, low fat hard cheese, hard cheese, soft cheese, blue cheese, low fat cheese spread, cheese spread, cottage cheese, feta, mozzarella, goats cheese, other cheese		
15.egg	whole egg, omelette, eggs in sandwiches, scotch egg, other egg	for each component, intakes below the lowest quintile score 5	for each component, intakes below the highest quintile score 1
16.fish or seafood	tinned tuna, oily fish, breaded fish, battered fish, white fish, prawns, lobster/crab, shellfish, other fish		
17.meat	sausage, beef, pork, lamb, crumbed or deep-fried poultry, poultry, bacon, ham, liver, other meat		
18. miscellaneous animal-based foods	pizza, chowder or cream soup		
total score		90	18

[a] Each component is divided into quintiles of consumption, and each quintile is assigned a score of 5 to 1.

Supplementary Table S6. Components and scoring criteria of the Healthy Eating Index 2015 (HEI-2015).

Components	Food	Max score [a]	Criteria for maximum score	Score of 0
1.total fruits	stewed fruit, prune, dried fruit, mixed fruit, apple, banana, berry, cherry, grapefruit, grape, mango, melon, orange, satsuma, peach/nectarine, pear, pineapple, plum, other fruit intake	5	≥0.8 cup eq. per 1,000 kcal	no intake
2.whole fruits	fruit juice, stewed fruit, prune, dried fruit, mixed fruit, apple, banana, berry, cherry,<br>grapefruit, grape, mango, melon, orange, satsuma, peach/nectarine, pear, pineapple, plum, other fruit	5	≥0.4 cup eq. per 1,000 kcal	no intake
3.total vegetables	baked bean, pulses, coleslaw, side salad, avocado, broad bean, green bean, beetroot, broccoli, butternut squash, cabbage/kale, carrot, cauliflower, celery, courgette, cucumber, garlic, leek, lettuce, mushroom, onion, parsnip, pea, sweet pepper, spinach, sprouts, sweetcorn, sweet potato, fresh tomato, tinned tomato, turnip/swede, watercress, other vegetables	5	≥1.1 cup eq. per 1,000 kcal	no intake
4.greens and beans	coleslaw, broccoli, celery, courgette, cucumber, leek, lettuce, spinach, sprouts, watercress, other vegetables, baked bean, pulses, broad bean, green bean, pea	5	≥0.2 cup eq. per 1,000 kcal	no intake
5.whole grains	muesli, oat crunch, sweetened cereal, plain cereal, bran cereal, whole-wheat cereal,<br>other cereal, oatcakes, whole-meal pasta, brown rice, couscous	10	≥1.5 oz eq. per 1,000 kcal	no intake
6.dairy	milk, flavoured milk, low fat hard cheese, hard cheese, soft cheese, blue cheese, low fat cheese spread, cheese spread, cottage cheese, feta, mozzarella, goats cheese, other cheese, milk-based pudding, other milk-based pudding, yogurt	10	≥1.3 cup eq. per 1,000 kcal	no intake
7.total protein foods	whole egg, scotch egg, other egg, sausage, beef, pork, lamb, crumbed or deep-fried poultry, poultry, bacon, ham, liver, other meat, tinned tuna, oily fish, breaded fish, battered fish, white fish, prawns, lobster/crab, shellfish, other fish, tofu, quorn, other vegetarian alternative, salted peanuts, unsalted peanuts, salted nuts, unsalted nuts, seeds, baked bean, pulses, broad bean, green bean, pea	5	≥2.5 oz eq. per 1,000 kcal	no intake
8.seafood and plant proteins	tinned tuna, oily fish, breaded fish, battered fish, white fish, prawns, lobster/crab, shellfish, other fish, vegetarian sausages/burgers, tofu, quorn, other vegetarian alternative, salted peanuts, unsalted peanuts, salted nuts, unsalted nuts, seeds, baked bean, pulses, broad bean, green bean, pea	5	≥0.8 oz eq. per 1,000 kcal	no intake

9.fatty acids	monounsaturated fatty acids, polyunsaturated fatty acids, saturated fatty acids	10	(MUFA +PUFA) /SFA ≥2.5	(MUFA + PUFA)/SFA≤1.2
10.refined grains	porridge, sliced bread, baguette, bap, bread roll, naan bread, garlic bread, crispbread, oatcakes, other bread, white pasta, whole-meal pasta, white rice, sushi, snackpot, other grain, double crust pastry, single crust pastry, crumble, pizza, pancake, scotch pancake, cake, doughnut, other dessert	10	≤1.8 oz eq. per 1,000 kcal	≥4.3 oz eq. per 1,000 kcal
11.sodium	sodium	10	≤1.1 g per 1,000 kcal	≥2.0 g per 1,000 kcal
12.added sugars	added sugars and preserves	10	≤6.5% of energy	≥26% of energy
13.saturated fats	saturated fat	10	≤8% of energy	≥16% of energy
total score			100	0

Abbreviations: MUFA, monounsaturated fatty acid; PUFA, polyunsaturated fatty acid; SFA, saturated fatty acid; eq., equivalents.
[a] Intakes between maximum score standard and zero points are scored proportionally.

Supplementary Table S7. Components and scoring criteria of EAT-Lancet Score.

Components	Food	Score for 1 point
1.whole grains	muesli, oat crunch, sweetened cereal, plain cereal, bran cereal, whole-wheat cereal, other cereal, sliced bread, baguette, bap, bread roll, naan bread, garlic bread, crispbread, oatcakes, other bread, cereal bar	≤ 464 g/day
2.vegetables	vegetable pieces, coleslaw, side salad, avocado, beetroot, broccoli, butternut squash, cabbage/kale, carrot, cauliflower, celery, courgette, cucumber, garlic, leek, lettuce, mushroom, other vegetables	≥ 200 g/day
3.fruits	squash, orange juice, grapefruit juice, pure fruit/vegetable juice, fruit smoothie, stewed fruit, prune, dried fruit, mixed fruit, apple, banana, berry, cherry, grapefruit, other fruit	≥ 100 g/day
4.tubers and starchy vegetables	fried potatoes, boiled/baked potatoes, mashed potato, crisp	≤ 100 g/day
5.dry beans, lentils, peas	baked bean, pulses, broad bean, green bean, pea	≤100g/day
6.soy foods	vegetarian sausages/burgers, tofu	≤50g/day
7.peanuts or treenuts	salted peanuts, unsalted peanuts, salted nuts, unsalted nuts, seeds	≥25g/day
8.dairy foods	low fat hard cheese, hard cheese, soft cheese, blue cheese, low fat cheese spread, cheese spread, cottage cheese, feta, mozzarella, goats cheese, other cheese, milk-based pudding, other milk-based pudding, dairy smoothie, milk, flavoured milk, yogurt	≤500g/day
9.added sugar	sugar added to coffee, sugar added to tea, sugar added to cereal	≤31g/day
10.beef, lamb, pork	beef, lamb, pork, bacon, ham	≤28g/day
11.poultry	crumbed or deep-fried poultry, poultry	≤58g/day
12.eggs	whole egg, omelette, eggs in sandwiches, scotch egg, other egg	≤25g/day
13.fish	tinned tuna, oily fish, breaded fish, battered fish, white fish, prawns, lobster/crab, shellfish, other fish	≤100g/day
14.added fats	monounsaturated fatty acids, polyunsaturated fatty acids, saturated fat	≥0.8 for unsaturated: saturated fat ratio
total score		14

Supplementary Table S8. Single-nucleotide polymorphisms used to construct the genetic risk score for cholelithiasis

ID	Chr	Position	Effect allele	Other allele	P value	EAF	Effect weight
rs62140201	2	42214198	A	G	2.97E-06	0.017823	0.138004
rs77273414	2	44062784	A	G	3.61E-08	0.017216	-0.17926
rs4401454	4	95741658	G	A	8.52E-08	0.194986	0.053809
rs35093039	5	24061216	A	G	2.54E-06	0.166293	0.050292
rs78488360	7	52968812	T	C	2.2E-06	0.020549	-0.13876
rs10832491	11	15652089	A	C	2.44E-06	0.077328	-0.07244
rs11020842	11	94317202	A	G	3.65E-06	0.03732	0.097566
rs4765873	12	2109251	T	C	2.4E-06	0.091698	0.064969
rs41276914	15	90347338	C	T	6.88E-07	0.074388	-0.07726
rs4468717	18	3457606	T	C	2.61E-06	0.138178	-0.056
rs516246	19	49206172	C	T	1.51E-08	0.625012	-0.04653
rs2618566	20	17844684	G	T	1.84E-08	0.32278	-0.04881
rs1800961	20	43042364	T	C	6.74E-68	0.045473	0.313022

Supplementary Table S9. Baseline characteristics of participants, according to the quartiles of MED.

**Characteristics**	**Q1**	**Q2**	**Q3**	**Q4**	Overall
	(N=13,970)	(N=28,365)	(N=37,987)	(N=75,001)	(N=155,323)
**Mean (SD) Age, years**	56.5 (7.92)	56.2 (7.90)	56.3 (7.89)	56.5 (7.72)	56.4 (7.81)
**Gender, N (%)**					
Female	6,029 (43.20)	14,018 (49.40)	20,026 (52.70)	42,754 (57.00)	82,827 (53.30)
Male	7,941 (56.80)	14,347 (50.60)	17,961 (47.30)	32,247 (43.00)	72,496 (46.70)
**Mean (SD) Index of multiple deprivation**	15.20 (12.40)	15.30 (12.40)	14.90 (12.10)	14.50 (11.70)	14.80 (12.00)
**Education level, N (%)**					
A levels/AS levels or equivalent	1,801 (12.90)	3,589 (12.70)	5,085 (13.40)	10,100 (13.50)	20,575 (13.20)
College or University degree	4,967 (35.60)	10,147 (35.80)	14,809 (39.00)	34,328 (45.80)	64,251 (41.40)
CSEs or equivalent	738 (5.30)	1,549 (5.50)	1,807 (4.80)	2,600 (3.50)	6,694 (4.30)
NVQ or HND or HNC or equivalent	921 (6.60)	1,793 (6.30)	2,205 (5.80)	3,783 (5.00)	8,702 (5.60)
O levels/GCSEs or equivalent	3,284 (23.50)	6,681 (23.60)	8,509 (22.40)	14,891 (19.90)	33,365 (21.50)
**Health rate, N (%)**					
Excellent	2,440 (17.50)	5,047 (17.80)	7,219 (19.00)	16,572 (22.10)	31,278 (20.10)
Good	8,352 (59.80)	16,971 (59.80)	22,691 (59.70)	45,121 (60.20)	93,135 (60.00)
Poor	466 (3.30)	982 (3.50)	1,190 (3.10)	1,808 (2.40)	4,446 (2.90)
**Long-standing illness, N (%)**	4,138 (29.60)	8,393 (29.60)	11,238 (29.60)	21,098 (28.10)	44,867 (28.90)
**Multivitamin use, N (%)**	1,824 (13.10)	3,838 (13.50)	5,346 (14.10)	11,836 (15.80)	22,844 (14.70)
**Intake of mineral supplements, N (%)**	2,862 (20.50)	5,806 (20.50)	8,110 (21.30)	17,359 (23.10)	34,137 (22.00)
**Mean (SD) body mass index, kg/m^2**	27.30 (4.42)	27.20 (4.57)	27.10 (4.60)	26.50 (4.53)	26.90 (4.56)
**Smoking status, N (%)**					
Current	1,405 (10.10)	2,640 (9.30)	3,047 (8.00)	4,482 (6.00)	11,574 (7.50)
Never	7,586 (54.30)	15,741 (55.50)	21,462 (56.50)	43,325 (57.80)	88,114 (56.70)
Previous	4,937 (35.30)	9,913 (34.90)	13,398 (35.30)	27,070 (36.10)	55,318 (35.60)
**Alcohol consumption, N (%)**					
Daily or almost daily	3,387 (24.20)	6,812 (24.00)	9,184 (24.20)	18,154 (24.20)	37,537 (24.20)

Never	690 (4.90)	1,494 (5.30)	1,931 (5.10)	4,054 (5.40)	8,169 (5.30)
Once or twice a week	3,650 (26.10)	7,219 (25.50)	9,622 (25.30)	18,517 (24.70)	39,008 (25.10)
One to three times a month	1,484 (10.60)	3,086 (10.90)	40,66 (10.70)	7,771 (10.40)	16,407 (10.60)
Special occasions only	1,154 (8.30)	2,597 (9.20)	3,372 (8.90)	6,431 (8.60)	13,554 (8.70)
Three or four times a week	3,602 (25.80)	7,147 (25.20)	9,799 (25.80)	20,056 (26.70)	40,604 (26.10)
**Physical activity, N (%)**					
high	4,293 (30.70)	8,674 (30.60)	11,999 (31.60)	26,158 (34.90)	51,124 (32.90)
low	2,352 (16.80)	4,641 (16.40)	5,810 (15.30)	10,159 (13.50)	22,962 (14.80)
moderate	4,733 (33.90)	9,703 (34.20)	13,309 (35.00)	26,667 (35.60)	54,412 (35.00)

Abbreviations: A levels/AS levels, advanced levels/advanced subsidiary levels; O levels/GCSE, ordinary levels/general certificate of secondary education; CSE, certificate of secondary education; NVQ,national vocational qualification; HND, higher national diploma; HNC, higher national certificate.

Supplementary Table S10. Baseline characteristics of participants, according to the quartiles of aMED.

Characteristics	Q1	Q2	Q3	Q4	Overall
	(N=35,413)	(N=35,662)	(N=35,430)	(N=48,818)	(N=155,323)
**Mean (SD) Age, years**	56.10 (7.92)	56.20 (7.90)	56.60 (7.78)	56.60 (7.69)	56.40 (7.81)
Gender, N (%)					
Female	17,527 (49.50)	18,425 (51.70)	18,942 (53.50)	27,933 (57.20)	82,827 (53.30)
Male	17,886 (50.5)	17,237 (48.30)	16,488 (46.50)	20,885 (42.80)	72,496 (46.70)
**Mean (SD) Index of multiple deprivation**	15.70 (12.70)	15.20 (12.30)	14.50 (11.70)	14.00 (11.30)	14.80 (12.00)
**Education level, N (%)**					
A levels/AS levels or equivalent	4,531 (12.80)	4,708 (13.20)	4,807 (13.60)	6,529 (13.40)	20,575 (13.20)
College or University degree	11,853 (33.50)	13,445 (37.70)	14,995 (42.30)	23,958 (49.10)	64,251 (41.40)
CSEs or equivalent	2,057 (5.80)	1,771 (5.00)	1,434 (4.00)	1,432 (2.90)	6,694 (4.30)
NVQ or HND or HNC or equivalent	2,306 (6.50)	2,138 (6.00)	1,937 (5.50)	2,321 (4.80)	8,702 (5.60)
O levels/GCSEs or equivalent	8,558 (24.20)	8,247 (23.10)	7,450 (21.00)	9,110 (18.70)	33,365 (21.50)
**Health rate, N (%)**					
Excellent	5,938 (16.80)	6,635 (18.60)	7,289 (20.60)	11,416 (23.40)	31,278 (20.10)
Good	20,920 (59.10)	21,359 (59.90)	21,442 (60.50)	29,414 (60.30)	93,135 (60.00)
Poor	1,356 (3.80)	1,189 (3.30)	882 (2.50)	1,019 (2.10)	4,446 (2.90)
**Long-standing illness, N (%)**	10,770 (30.40)	10,508 (29.50)	10,137 (28.60)	13,452 (27.60)	4,4867 (28.90)
**Multivitamin use, N (%)**	4,671 (13.20)	4,994 (14.00)	5,228 (14.80)	7,951 (16.30)	22,844 (14.70)
**Intake of mineral supplements, N (%)**	7,120 (20.10)	7,533 (21.10)	7,889 (22.30)	11,595 (23.80)	34,137 (22.00)
**Mean (SD) body mass index, kg/m^2**	27.40 (4.66)	27.10 (4.57)	26.80 (4.51)	26.30 (4.44)	26.90(4.56)
**Smoking status, N (%)**					
Current	3,784 (10.70)	2,993 (8.40)	2,430 (6.90)	2,367 (4.80)	11,574 (7.50)
Never	18,908 (53.40)	19,831 (55.60)	20,318 (57.30)	29,057 (59.50)	88,114 (56.70)
Previous	12,624 (35.60)	12,767 (35.80)	12,620 (35.60)	17,307 (35.50)	55,318 (35.60)
**Alcohol consumption, N (%)**					
Daily or almost daily	8,788 (24.80)	8,907 (25.00)	8,902 (25.10)	10,940 (22.40)	37,537 (24.20)
Never	2,105 (5.90)	1,933 (5.40)	1,877 (5.30)	22,54 (4.60)	8,169 (5.30)

Once or twice a week	8,711 (24.60)	8,711 (24.40)	8,733 (24.60)	12,853 (26.30)	39,008 (25.10)
One to three times a month	3,818 (10.80)	3,902 (10.90)	3,704 (10.50)	4,983 (10.20)	16,407 (10.60)
Special occasions only	3,468 (9.80)	3,246 (9.10)	3,037 (8.60)	3,803 (7.80)	13,554 (8.70)
Three or four times a week	8,506 (24.00)	8,954 (25.10)	9,169 (25.90)	13,975 (28.60)	40,604 (26.10)
**Physical activity, N (%)**					
high	10,770 (30.40)	11,377 (31.90)	11,574 (32.70)	17,403 (35.60)	51,124 (32.90)
low	5,839 (16.50)	5,546 (15.60)	5,171 (14.60)	6,406 (13.10)	22,962 (14.80)
moderate	11,891 (33.60)	12,258 (34.40)	12,665 (35.70)	17,598 (36.00)	54,412 (35.00)

Abbreviations: A levels/AS levels, advanced levels/advanced subsidiary levels; O levels/GCSE, ordinary levels/general certificate of secondary education; CSE, certificate of secondary education; NVQ,national vocational qualification; HND, higher national diploma; HNC, higher national certificate.

Supplementary Table S11. Baseline characteristics of participants, according to the quartiles of PDI.

Characteristics	Q1	Q2	Q3	Q4	Overall
	(N=36,741)	(N=33,623)	(N=40,759)	(N=44,200)	(N=155,323)
**Mean (SD) Age, years**	56.20 (7.90)	56.30 (7.87)	56.50 (7.80)	56.50 (7.71)	56.40 (7.81)
**Gender, N (%)**					
Female	18,307 (49.80)	17,374 (51.70)	21,885 (53.70)	25,261 (57.20)	82,827 (53.30)
Male	18,434 (50.20)	16,249 (48.30)	18,874 (46.30)	18,939 (42.80)	72,496 (46.70)
**Mean (SD) Index of multiple deprivation**	15.80 (12.70)	15.00 (12.20)	14.50 (11.60)	14.10 (11.40)	14.80 (12.00)
**Education level, N (%)**					
A levels/AS levels or equivalent	4,687 (12.80)	4,485 (13.30)	5,414 (13.30)	5,989 (13.50)	20,575 (13.20)
College or University degree	12,493 (34.00)	12,937 (38.50)	17,439 (42.80)	21,382 (48.40)	64,251 (41.40)
CSEs or equivalent	2,147 (5.80)	1,631 (4.90)	1,576 (3.90)	1,340 (3.00)	6,694 (4.30)
NVQ or HND or HNC or equivalent	2,382 (6.50)	2,008 (6.00)	2,189 (5.40)	2,123 (4.80)	8,702 (5.60)
O levels/GCSEs or equivalent	8,784 (23.90)	7,591 (22.60)	8,668 (21.30)	8,322 (18.80)	33,365 (21.50)
**Health rate, N (%)**					
Excellent	6,164 (16.80)	6,484 (19.30)	8,306 (20.40)	10,324 (23.40)	31,278 (20.10)
Good	21,902 (59.60)	20,042 (59.60)	24,655 (60.50)	26,536 (60.00)	93,135 (60.00)
Poor	1,328 (3.60)	1,045 (3.10)	1,053 (2.60)	1,020 (2.30)	4,446 (2.90)
**Long-standing illness, N (%)**	10,953 (29.80)	9,756 (29.00)	11,792 (28.90)	12,366 (28.00)	4,4867 (28.90)
**Multivitamin use, N (%)**	4,882 (13.30)	4,862 (14.50)	6,015 (14.80)	7,085 (16.00)	22,844 (14.70)
**Intake of mineral supplements, N (%)**	7,482 (20.40)	7,130 (21.20)	8,977 (22.00)	10,548 (23.90)	34,137 (22.00)
**Mean (SD) body mass index, kg/m^2**	27.50 (4.74)	27.00 (4.55)	26.80 (4.48)	26.30 (4.40)	26.90 (4.56)
**Smoking status, N (%)**					
Current	3,758 (10.20)	2,761 (8.20)	2,741 (6.70)	2,314 (5.20)	11,574 (7.50)
Never	19,380 (52.70)	18,841 (56.00)	23,474 (57.60)	26,419 (59.80)	88,114 (56.70)
Previous	13,504 (36.80)	11,944 (35.50)	14,467 (35.50)	15,403 (34.80)	55,318 (35.60)
**Alcohol consumption, N (%)**					
Daily or almost daily	9,791 (26.60)	8,274 (24.60)	9,606 (23.60)	9,866 (22.30)	37,537 (24.20)
Never	1,703 (4.60)	1,759 (5.20)	2,186 (5.40)	2,521 (5.70)	8,169 (5.30)

Once or twice a week	9,099 (24.80)	8,449 (25.10)	10,287 (25.20)	11,173 (25.30)	39,008 (25.10)
One to three times a month	3,716 (10.10)	3,530 (10.50)	4,346 (10.70)	4,815 (10.90)	16,407 (10.60)
Special occasions only	3,091 (8.40)	2,920 (8.70)	3,597 (8.80)	3,946 (8.90)	13,554 (8.70)
Three or four times a week	9,326 (25.40)	8,677 (25.80)	10,730 (26.30)	11,871 (26.90)	40,604 (26.10)
**Physical activity, N (%)**					
high	11,280 (30.70)	10,694 (31.80)	13,483 (33.10)	15,667 (35.40)	51,124 (32.90)
low	6,105 (16.60)	5,100 (15.20)	6,005 (14.70)	57,52 (13.00)	22,962 (14.80)
moderate	12,289 (33.40)	11,781 (35.00)	14,412 (35.40)	15,930 (36.00)	54,412 (35.00)

Abbreviations: A levels/AS levels, advanced levels/advanced subsidiary levels; O levels/GCSE, ordinary levels/general certificate of secondary education; CSE, certificate of secondary education; NVQ,national vocational qualification; HND, higher national diploma; HNC, higher national certificate.

Supplementary Table S12. Baseline characteristics of participants, according to the quartiles of hPDI.

**Characteristics**	**Q1**	**Q2**	**Q3**	**Q4**	Overall
	(N=33,034)	(N=39,501)	(N=42,980)	(N=39,808)	(N=155,323)
**Mean (SD) Age, years**	55.40 (8.02)	56.30 (7.81)	56.60 (7.79)	57.00 (7.59)	56.40 (7.81)
**Gender, N (%)**					
Female	14,585 (44.20)	19,957 (50.50)	23,631 (55.00)	24,654 (61.90)	82,827 (53.30)
Male	18,449 (55.80)	19,544 (49.50)	19,349 (45.00)	15,154 (38.10)	72,496 (46.70)
**Mean (SD) Index of multiple deprivation**	15.30 (12.30)	14.80 (12.00)	14.80 (12.00)	14.50 (11.70)	14.80 (12.00)
**Education level, N (%)**					
A levels/AS levels or equivalent	4,618 (14.00)	5,208 (13.20)	5,735 (13.30)	5,014 (12.60)	20,575 (13.20)
College or University degree	13,597 (41.20)	15,901 (40.30)	17,491 (40.70)	17,262 (43.40)	64,251 (41.40)
CSEs or equivalent	1,534 (4.60)	1,812 (4.60)	1,852 (4.30)	1,496 (3.80)	6,694 (4.30)
NVQ or HND or HNC or equivalent	1,926 (5.80)	2,324 (5.90)	2,388 (5.60)	2,064 (5.20)	8,702 (5.60)
O levels/GCSEs or equivalent	7,237 (21.90)	8,632 (21.90)	9,320 (21.70)	8,176 (20.50)	33,365 (21.50)
**Health rate, N (%)**					
Excellent	6,104 (18.50)	7,809 (19.80)	8,568 (19.90)	8,797 (22.10)	31,278 (20.10)
Good	19,426 (58.80)	23,642 (59.90)	26,059 (60.60)	24,008 (60.30)	93,135 (60.00)
Poor	1,140 (3.50)	1,178 (3.00)	1,188 (2.80)	940 (2.40)	4,446 (2.90)
**Long-standing illness, N (%)**	10,244 (31.00)	11,455 (29.00)	12,189 (28.40)	10,979 (27.60)	44,867 (28.90)
**Multivitamin use, N (%)**	4,412 (13.40)	5,606 (14.20)	6,259 (14.60)	6,567 (16.50)	2,2844 (14.70)
**Intake of mineral supplements, N (%)**	6,789 (20.60)	8,366 (21.20)	9,424 (21.90)	9,558 (24.00)	34,137 (22.00)
**Mean (SD) body mass index, kg/m^2**	27.60 (4.85)	27.10 (4.610)	26.70 (4.40)	26.20 (4.30)	26.90 (4.56)
**Smoking status, N (%)**					
Current	2,568 (7.80)	3,098 (7.80)	3,264 (7.60)	2,644 (6.60)	11,574 (7.50)
Never	19,065 (57.70)	22,401 (56.70)	24,161 (56.20)	22,487 (56.50)	88,114 (56.70)
Previous	11,337 (34.30)	13,930 (35.30)	15,464 (36.00)	14,587 (36.60)	55,318 (35.60)
**Alcohol consumption, N (%)**					
Daily or almost daily	7,902 (23.90)	9,511 (24.10)	10,524 (24.50)	9,600 (24.10)	37,537 (24.20)
Never	1,820 (5.50)	2,055 (5.20)	2,171 (5.10)	2,123 (5.30)	8,169 (5.30)

Once or twice a week	8,292 (25.10)	10,129 (25.60)	10,797 (25.10)	97,90 (24.60)	39,008 (25.10)
One to three times a month	3,689 (11.20)	4,203 (10.60)	4,456 (10.40)	4,059 (10.20)	16,407 (10.60)
Special occasions only	2,938 (8.90)	3,367 (8.50)	3,621 (8.40)	3,628 (9.10)	13,554 (8.70)
Three or four times a week	8,383 (25.40)	10,225 (25.90)	11,397 (26.50)	10,599 (26.60)	40,604 (26.10)
**Physical activity, N (%)**					
high	10,039 (30.40)	12,461 (31.50)	1,4291 (33.30)	14,333 (36.00)	51,124 (32.90)
low	5,778 (17.50)	6,079 (15.40)	6,236 (14.50)	4,869 (12.20)	22,962 (14.80)
moderate	11,868 (35.90)	14,000 (35.40)	14,884 (34.60)	13,660 (34.30)	54,412 (35.00)

Abbreviations: A levels/AS levels, advanced levels/advanced subsidiary levels; O levels/GCSE, ordinary levels/general certificate of secondary education; CSE, certificate of secondary education; NVQ,national vocational qualification; HND, higher national diploma; HNC, higher national certificate.

Supplementary Table S13. Baseline characteristics of participants, according to the quartiles of uPDI.

Characteristics	Q1	Q2	Q3	Q4	Overall
	(N=32,766)	(N=40,917)	(N=41,961)	(N=39,679)	(N=155,323)
**Mean (SD) Age, years**	57.00 (7.56)	56.70 (7.76)	56.40 (7.82)	55.50 (8.00)	56.40 (7.81)
**Gender, N (%)**					
Female	20,570 (62.80)	22,867 (55.90)	21,520 (51.30)	17,870 (45.00)	82,827 (53.30)
Male	12,196 (37.20)	18,050 (44.10)	20,441 (48.70)	21,809 (55.00)	72,496 (46.70)
**Mean (SD) Index of multiple deprivation**	14.50 (11.60)	14.70 (11.90)	14.70 (12.00)	15.20 (12.30)	14.80 (12.00)
**Education level, N (%)**					
A levels/AS levels or equivalent	4,113 (12.60)	5,422 (13.30)	5,513 (13.10)	5,527 (13.90)	20,575 (13.20)
College or University degree	14,331 (43.70)	16,747 (40.90)	16,870 (40.20)	16,303 (41.10)	64,251 (41.40)
CSEs or equivalent	1,234 (3.80)	1,716 (4.20)	1,915 (4.60)	1,829 (4.60)	6,694 (4.30)
NVQ or HND or HNC or equivalent	1,664 (5.10)	2,298 (5.60)	2,473 (5.90)	2,267 (5.70)	8,702 (5.60)
O levels/GCSEs or equivalent	6,670 (20.40)	8,831 (21.60)	9,153 (21.80)	8,711 (22.00)	33,365 (21.50)
**Health rate, N (%)**					
Excellent	7,313 (22.30)	8,306 (20.30)	8,232 (19.60)	7,427 (18.70)	31,278 (20.10)
Good	19,808 (60.50)	24,778 (60.60)	25,177 (60.00)	23,372 (58.90)	93,135 (60.00)
Poor	749 (2.30)	1,102 (2.70)	1,253 (3.00)	1,342 (3.40)	4,446 (2.90)
**Long-standing illness, N (%)**	8,956 (27.30)	11,553 (28.20)	12,156 (29.00)	12,202 (30.80)	44,867 (28.90)
**Multivitamin use, N (%)**	5,498 (16.80)	6,047 (14.80)	5,984 (14.30)	5,315 (13.40)	22,844 (14.70)
**Intake of mineral supplements, N (%)**	7,972 (24.30)	9,041 (22.10)	8,956 (21.30)	8,168 (20.60)	34,137 (22.00)
**Mean (SD) body mass index, kg/m^2**	26.20 (4.29)	26.60 (4.37)	27.00 (4.59)	27.50 (4.82)	26.90 (4.56)
**Smoking status, N (%)**					
Current	2,152 (6.60)	3,063 (7.50)	3,251 (7.70)	3,108 (7.80)	11,574 (7.50)
Never	18,485 (56.40)	23,086 (56.40)	23,674 (56.40)	22,869 (57.60)	88,114 (56.70)
Previous	12,059 (36.80)	14,683 (35.90)	14,949 (35.60)	13,627 (34.30)	55,318 (35.60)
**Alcohol consumption, N (%)**					
Daily or almost daily	7,857 (24.00)	10,048 (24.60)	10,156 (24.20)	9,476 (23.90)	37,537 (24.20)
Never	1,756 (5.40)	2,068 (5.10)	2,180 (5.20)	2,165 (5.50)	8,169 (5.30)

Once or twice a week	8,004 (24.40)	10,259 (25.10)	10,692 (25.50)	10,053 (25.30)	39,008 (25.10)
One to three times a month	3,341 (10.20)	4,181 (10.20)	4,501 (10.70)	4,384 (11.00)	16,407 (10.60)
Special occasions only	3,023 (9.20)	3,464 (8.50)	3,592 (8.60)	3,475 (8.80)	13,554 (8.70)
Three or four times a week	8,778 (26.80)	10,883 (26.60)	10,828 (25.80)	10,115 (25.50)	40,604 (26.10)
**Physical activity, N (%)**					
high	11,938 (36.40)	13,661 (33.40)	13,415 (32.00)	12,110 (30.50)	51,124 (32.90)
low	3,914 (11.90)	5,831 (14.30)	6,403 (15.30)	6,814 (17.20)	22,962 (14.80)
moderate	11,216 (34.20)	14,180 (34.70)	14,774 (35.20)	14,242 (35.90)	54,412 (35.00)

Abbreviations: A levels/AS levels, advanced levels/advanced subsidiary levels; O levels/GCSE, ordinary levels/general certificate of secondary education; CSE, certificate of secondary education; NVQ,national vocational qualification; HND, higher national diploma; HNC, higher national certificate.

Supplementary Table S14. Baseline characteristics of participants, according to the quartiles of HEI-2015.

Characteristics	Q1	Q2	Q3	Q4	Overall
	(N=38,831)	(N=38,830)	(N=38,831)	(N=38,831)	(N=155,323)
**Mean (SD) Age, years**	55.60 (8.08)	56.20 (7.92)	56.60 (7.69)	57.10 (7.47)	56.40 (7.81)
**Gender, N (%)**					
Female	17,536 (45.20)	19,690 (50.70)	21,231 (54.70)	24,370 (62.80)	82,827 (53.30)
Male	21,295 (54.80)	19,140 (49.30)	17,600 (45.30)	14,461 (37.20)	72,496 (46.70)
**Mean (SD) Index of multiple deprivation**	15.90 (12.80)	14.80 (12.00)	14.30 (11.70)	14.10 (11.20)	14.80 (12.00)
**Education level, N (%)**					
A levels/AS levels or equivalent	4,933 (12.70)	5,208 (13.40)	5,152 (13.30)	5,282 (13.60)	20,575 (13.20)
College or University degree	13,658 (35.20)	15,704 (40.40)	17,004 (43.80)	17,885 (46.10)	64,251 (41.40)
CSEs or equivalent	2,216 (5.70)	1,773 (4.60)	1,518 (3.90)	1,187 (3.10)	6,694 (4.30)
NVQ or HND or HNC or equivalent	2,540 (6.50)	2,168 (5.60)	2,115 (5.40)	1,879 (4.80)	8,702 (5.60)
O levels/GCSEs or equivalent	9,073 (23.40)	8,415 (21.70)	8,044 (20.70)	7,833 (20.20)	33,365 (21.50)
**Health rate, N (%)**					
Excellent	6,659 (17.10)	7,529 (19.40)	8,124 (20.90)	8,966 (23.10)	31,278 (20.10)
Good	22,811 (58.70)	23,450 (60.40)	23,469 (60.40)	23,405 (60.30)	93,135 (60.00)
Poor	1,500 (3.90)	1,130 (2.90)	970 (2.50)	846 (2.20)	4,446 (2.90)
**Long-standing illness, N (%)**	11,687 (30.10)	11,179 (28.80)	11,160 (28.70)	10,841 (27.90)	44,867 (28.90)
**Multivitamin use, N (%)**	5,007 (12.90)	5,443 (14.00)	5,780 (14.90)	6,614 (17.00)	22,844 (14.70)
**Intake of mineral supplements, N (%)**	7,770 (20.00)	8,265 (21.30)	8,706 (22.40)	9,396 (24.20)	34,137 (22.00)
**Mean (SD) body mass index, kg/m^2**	27.20 (4.60)	27.00 (4.56)	26.80 (4.53)	26.50 (4.50)	26.90 (4.56)
**Smoking status, N (%)**					
Current	4,257 (11.00)	2,958 (7.60)	2,439 (6.30)	1,920 (4.90)	11,574 (7.50)
Never	20,906 (53.80)	22,066 (56.80)	22,471 (57.90)	22,671 (58.40)	88,114 (56.70)
Previous	13,558 (34.90)	13,735 (35.40)	13,854 (35.70)	14,171 (36.50)	55,318 (35.60)
**Alcohol consumption, N (%)**					
Daily or almost daily	9,178 (23.60)	9,611 (24.80)	9,660 (24.90)	9,088 (23.40)	37,537 (24.20)
Never	2,179 (5.60)	2,058 (5.30)	1,871 (4.80)	2,061 (5.30)	8,169 (5.30)

Once or twice a week	9,915 (25.50)	9,716 (25.00)	9,669 (24.90)	9,708 (25.00)	39,008 (25.10)
One to three times a month	4,269 (11.00)	4,032 (10.40)	4,065 (10.50)	4,041 (10.40)	16,407 (10.60)
Special occasions only	3,687 (9.50)	3,197 (8.20)	3,219 (8.30)	3,451 (8.90)	13,554 (8.70)
Three or four times a week	9,584 (24.70)	10,211 (26.30)	10,337 (26.60)	10,472 (27.00)	40,604 (26.10)
**Physical activity, N (%)**					
high	12,051 (31.00)	12,513 (32.20)	12,789 (32.90)	13,771 (35.50)	51,124 (32.90)
low	6,250 (16.10)	5,926 (15.30)	5,765 (14.80)	5,021 (12.90)	22,962 (14.80)
moderate	13,140 (33.80)	13,580 (35.00)	13,859 (35.70)	13,833 (35.6)	54,412 (35.00)

Abbreviations: A levels/AS levels, advanced levels/advanced subsidiary levels; O levels/GCSE, ordinary levels/general certificate of secondary education; CSE, certificate of secondary education; NVQ, national vocational qualification; HND, higher national diploma; HNC, higher national certificate.

Supplementary Table S15. Baseline characteristics of participants, according to the quartiles of EAT-lancet score.

Characteristics	Q1	Q2	Q3	Q4	Overall
	(N=27,548)	(N=36,456)	(N=43,184)	(N=48,135)	(N=155,323)
**Mean (SD) Age, years**	57.00 (7.69)	56.60 (7.79)	56.30 (7.85)	55.90 (7.85)	56.40 (7.81)
**Gender, N (%)**					
Female	12,594 (45.70)	18,426 (50.50)	23,327 (54.00)	28,480 (59.20)	82,827 (53.30)
Male	14,954 (54.30)	18,030 (49.50)	19,857 (46.00)	19,655 (40.80)	72,496 (46.70)
**Mean (SD) Index of multiple deprivation**	15.10 (12.20)	14.80 (12.00)	14.80 (12.00)	14.60 (11.80)	14.80 (12.00)
**Education level, N (%)**					
A levels/AS levels or equivalent	3,771 (13.70)	4,885 (13.40)	5,615 (13.00)	6,304 (13.10)	20,575 (13.20)
College or University degree	10,969 (39.80)	14,457 (39.70)	17,460 (40.40)	21,365 (44.40)	64,251 (41.40)
CSEs or equivalent	1,166 (4.20)	1,614 (4.40)	1,909 (4.40)	2,005 (4.20)	6,694 (4.30)
NVQ or HND or HNC or equivalent	1,744 (6.30)	2,136 (5.90)	2,464 (5.70)	2,358 (4.90)	8,702 (5.60)
O levels/GCSEs or equivalent	5,966 (21.70)	8,094 (22.20)	9,473 (21.90)	9,832 (20.40)	33,365 (21.50)
**Health rate, N (%)**					
Excellent	5,203 (18.90)	7,109 (19.50)	8,770 (20.30)	10,196 (21.20)	31,278 (20.10)
Good	16,377 (59.40)	21,834 (59.90)	25,920 (60.00)	29,004 (60.30)	93,135 (60.00)
Poor	879 (3.20)	1,130 (3.10)	1,242 (2.90)	1,195 (2.50)	4,446 (2.90)
**Long-standing illness, N (%)**	8,587 (31.20)	10,652 (29.20)	12,297 (28.50)	13,331 (27.70)	44,867 (28.90)
**Multivitamin use, N (%)**	3,759 (13.60)	5,143 (14.10)	6,250 (14.50)	7,692 (16.00)	22,844 (14.70)
**Intake of mineral supplements, N (%)**	5,518 (20.00)	7,559 (20.70)	9,431 (21.80)	11,629 (24.20)	34,137 (22.00)
**Mean (SD) body mass index, kg/m^2**	27.40 (4.71)	27.10 (4.57)	26.90 (4.56)	26.40(4.40)	26.90 (4.56)
**Smoking status, N (%)**					
Current	2,396 (8.70)	2,848 (7.80)	3,236 (7.50)	3,094 (6.40)	11,574 (7.50)
Never	15,144 (55.00)	20,543 (56.40)	24,564 (56.90)	27,863 (57.90)	88,114 (56.70)
Previous	9,964 (36.20)	12,990 (35.60)	15,277 (35.40)	17,087 (35.50)	55,318 (35.60)
**Alcohol consumption, N (%)**					
Daily or almost daily	6,942 (25.20)	8,939 (24.50)	10,495 (24.30)	11,161 (23.20)	37,537 (24.20)
Never	1,418 (5.10)	1,853 (5.10)	2,233 (5.20)	2,665 (5.50)	8,169 (5.30)

Once or twice a week	6,777 (24.60)	9,196 (25.20)	10,938 (25.30)	12,097 (25.10)	39,008 (25.10)
One to three times a month	2,919 (10.60)	3,817 (10.50)	4,513 (10.50)	5,158 (10.70)	16,407 (10.60)
Special occasions only	2,394 (8.70)	3,153 (8.60)	3,758 (8.70)	4,249 (8.80)	13,554 (8.70)
Three or four times a week	7,091 (25.70)	9,487 (26.00)	11,234 (26.00)	12,792 (26.60)	40,604 (26.10)
**Physical activity, N (%)**					
high	8,580 (31.10)	11,585 (31.80)	14,114 (32.70)	16,845 (35.00)	51,124 (32.90)
low	4,519 (16.40)	5,726 (15.70)	6,369 (14.70)	6,348 (13.20)	22,962 (14.80)
moderate	9,843 (35.70)	12,790 (35.10)	15,043 (34.80)	16,736 (34.80)	54,412 (35.00)

Abbreviations: A levels/AS levels, advanced levels/advanced subsidiary levels; O levels/GCSE, ordinary levels/general certificate of secondary education; CSE, certificate of secondary education; NVQ, national vocational qualification; HND, higher national diploma; HNC, higher national certificate.

Supplementary Table S16. Subgroup analysis for the association between MED and risk of cholelithiasis incidence.

Subgroups	HR (95% CI)				*P* for interaction
	Q1	Q2	Q3	Q4	
**Age**					
<60 years	1.00 (ref)	1.05 (0.90-1.23)	1.08 (0.93-1.26)	0.96 (0.83-1.11)	0.426
≥60 years	1.00 (ref)	1.10 (0.94-1.29)	1.00 (0.86-1.17)	1.02 (0.88-1.18)	
**Gender**					
Female	1.00 (ref)	1.08 (0.92-1.25)	0.98 (0.85-1.14)	0.99 (0.86-1.14)	0.258
Male	1.00 (ref)	1.06 (0.90-1.26)	1.13 (0.96-1.33)	0.98 (0.84-1.14)	
**Education level**					
College or University degree	1.00 (ref)	1.07 (0.85-1.33)	1.14 (0.93-1.41)	1.02 (0.83-1.24)	0.479
Other	1.00 (ref)	1.07 (0.94-1.22)	0.99 (0.88-1.13)	0.97 (0.86-1.09)	
**Body mass index**					
<25 kg/m^2	1.00 (ref)	1.48 (1.12-1.94)	1.41 (1.08-1.84)	1.06 (0.82-1.37)	0.017
≥25 kg/m^2	1.00 (ref)	1.00 (0.89-1.14)	0.98 (0.87-1.11)	0.99 (0.89-1.11)	
**Drinker**					
No	1.00 (ref)	1.00 (0.79-1.27)	0.91 (0.73-1.15)	0.85 (0.68-1.05)	0.715
Yes	1.00 (ref)	1.10 (0.97-1.25)	1.09 (0.96-1.23)	1.04 (0.92-1.17)	
**Smoking status**					
Never	1.00 (ref)	1.02 (0.87-1.19)	1.03 (0.88-1.19)	0.95 (0.82-1.09)	
Previous	1.00 (ref)	1.14 (0.95-1.37)	1.07 (0.90-1.28)	1.07 (0.91-1.27)	0.657
Current	1.00 (ref)	1.07 (0.74-1.54)	1.02 (0.71-1.46)	0.87 (0.61-1.24)	
**Physical activity**					
Low	1.00 (ref)	0.96 (0.81-1.14)	0.99 (0.84-1.17)	0.95 (0.81-1.11)	
Moderate	1.00 (ref)	1.13 (0.92-1.38)	1.07 (0.88-1.29)	0.96 (0.80-1.16)	0.486
High	1.00 (ref)	1.20 (0.95-1.50)	1.08 (0.86-1.34)	1.08 (0.88-1.33)	

Estimated effects were calculated using Cox model stratified by age, sex, UK Biobank assessment centers, and additionally adjusted for overall health rating, long-standing illness, education, index of multiple deprivation, multivitamin use, mineral use, smoking status, alcohol consumption, physical activity and body mass index.

Supplementary Table S17. Subgroup analysis for the association between aMED and risk of cholelithiasis incidence.

Subgroups	HR (95% CI)				*P* for interaction
	Q1	Q2	Q3	Q4	
**Age**					
<60 years	1.00 (ref)	1.05 (0.94-1.17)	1.02 (0.91-1.14)	0.89 (0.79-0.99)	0.779
≥60 years	1.00 (ref)	1.00 (0.89-1.12)	1.01 (0.90-1.14)	0.91 (0.82-1.02)	
**Gender**					
Female	1.00 (ref)	0.98 (0.88-1.08)	1.00 (0.90-1.11)	0.93 (0.84-1.02)	0.258
Male	1.00 (ref)	1.10 (0.97-1.24)	1.04 (0.92-1.18)	0.86 (0.76-0.98)	
**Education level**					
College or University degree	1.00 (ref)	1.11 (0.95-1.30)	1.04 (0.89-1.21)	0.92 (0.80-1.06)	0.298
Other	1.00 (ref)	0.99 (0.90-1.08)	1.00 (0.91-1.10)	0.89 (0.81-0.98)	
**Body mass index**					
<25 kg/m^2	1.00 (ref)	1.01 (0.84-1.21)	0.96 (0.80-1.16)	0.78 (0.65-0.93)	0.245
≥25 kg/m^2	1.00 (ref)	1.02 (0.93-1.11)	1.03 (0.94-1.12)	0.94 (0.86-1.02)	
**Drinker**					
No	1.00 (ref)	0.98 (0.83-1.15)	1.02 (0.86-1.21)	0.72 (0.60-0.86)	0.008
Yes	1.00 (ref)	1.04 (0.95-1.14)	1.01 (0.92-1.11)	0.96 (0.88-1.05)	
**Smoking status**					
Never	1.00 (ref)	1.02 (0.91-1.14)	1.02 (0.91-1.14)	0.88 (0.79-0.98)	
Previous	1.00 (ref)	1.05 (0.92-1.19)	1.04 (0.91-1.19)	0.97 (0.85-1.10)	0.656
Current	1.00 (ref)	0.95 (0.73-1.24)	0.86 (0.64-1.16)	0.80 (0.59-1.10)	
**Physical activity**					
Low	1.00 (ref)	1.03 (0.91-1.17)	1.04 (0.92-1.18)	0.91 (0.81-1.04)	
Moderate	1.00 (ref)	1.01 (0.87-1.16)	0.99 (0.86-1.14)	0.87 (0.76-1.00)	0.513
High	1.00 (ref)	1.01 (0.87-1.19)	1.00 (0.86-1.18)	0.91 (0.78-1.05)	

Estimated effects were calculated using Cox model stratified by age, sex, UK Biobank assessment centers, and additionally adjusted for overall health rating, long-standing illness, education, index of multiple deprivation, multivitamin use, mineral use, smoking status, alcohol consumption, physical activity and body mass index.

Supplementary Table S18. Subgroup analysis for the association between PDI and risk of cholelithiasis incidence.

Subgroups	HR (95% CI)				*P* for interaction
	Q1	Q2	Q3	Q4	
**Age**					
<60 years	1.00 (ref)	1.01 (0.90-1.14)	1.01 (0.91-1.13)	1.00 (0.89-1.11)	0.049
≥60 years	1.00 (ref)	1.02 (0.90-1.14)	0.97 (0.87-1.09)	1.03 (0.92-1.15)	
**Gender**					
Female	1.00 (ref)	1.01 (0.91-1.12)	1.00 (0.90-1.11)	1.00 (0.90-1.11)	0.643
Male	1.00 (ref)	1.02 (0.89-1.15)	0.97 (0.85-1.10)	1.03 (0.90-1.16)	
**Education level**					
College or University degree	1.00 (ref)	1.05 (0.89-1.22)	1.03 (0.89-1.20)	1.03 (0.89-1.19)	0.397
Other	1.00 (ref)	1.00 (0.91-1.10)	0.97 (0.88-1.07)	1.00 (0.91-1.10)	
**Body mass index**					
<25 kg/m^2	1.00 (ref)	1.09 (0.90-1.31)	1.06 (0.88-1.26)	0.88 (0.74-1.06)	0.012
≥25 kg/m^2	1.00 (ref)	0.98 (0.89-1.07)	0.95 (0.87-1.04)	1.03 (0.94-1.12)	
**Drinker**					
No	1.00 (ref)	1.14 (0.96-1.36)	1.09 (0.92-1.29)	0.97 (0.81-1.16)	0.852
Yes	1.00 (ref)	0.99 (0.91-1.09)	0.98 (0.89-1.07)	1.04 (0.95-1.14)	
**Smoking status**					
Never	1.00 (ref)	1.06 (0.95-1.19)	1.02 (0.92-1.14)	1.02 (0.91-1.14)	
Previous	1.00 (ref)	0.98 (0.86-1.12)	0.99 (0.87-1.12)	1.01 (0.89-1.14)	0.734
Current	1.00 (ref)	0.84 (0.64-1.12)	0.78 (0.58-1.05)	1.06 (0.80-1.41)	
**Physical activity**					
Low	1.00 (ref)	1.05 (0.92-1.19)	1.05 (0.92-1.18)	1.04 (0.92-1.18)	
Moderate	1.00 (ref)	1.03 (0.89-1.19)	0.96 (0.84-1.11)	1.03 (0.90-1.18)	0.415
High	1.00 (ref)	0.96 (0.81-1.12)	0.96 (0.82-1.11)	0.94 (0.81-1.10)	

Estimated effects were calculated using Cox model stratified by age, sex, UK Biobank assessment centers, and additionally adjusted for overall health rating, long-standing illness, education, index of multiple deprivation, multivitamin use, mineral use, smoking status, alcohol consumption, physical activity and body mass index.

Supplementary Table S19. Subgroup analysis for the association between hPDI and risk of cholelithiasis incidence.

Subgroups	HR (95% CI)				*P* for interaction
	Q1	Q2	Q3	Q4	
**Age**					
<60 years	1.00 (ref)	1.03 (0.92-1.15)	1.01 (0.91-1.13)	0.98 (0.87-1.10)	0.644
≥60 years	1.00 (ref)	0.99 (0.88-1.11)	0.92 (0.81-1.03)	0.93 (0.83-1.05)	
**Gender**					
Female	1.00 (ref)	0.95 (0.85-1.05)	0.92 (0.83-1.02)	0.97 (0.87-1.08)	0.061
Male	1.00 (ref)	1.09 (0.96-1.23)	1.03 (0.91-1.17)	0.92 (0.81-1.06)	
**Education level**					
College or University degree	1.00 (ref)	1.07 (0.92-1.23)	0.98 (0.84-1.13)	0.98 (0.84-1.14)	0.942
Other	1.00 (ref)	0.98 (0.89-1.08)	0.97 (0.88-1.07)	0.96 (0.87-1.06)	
**Body mass index**					
<25 kg/m^2	1.00 (ref)	0.95 (0.79-1.15)	0.92 (0.77-1.11)	0.91 (0.75-1.09)	0.243
≥25 kg/m^2	1.00 (ref)	0.99 (0.91-1.08)	0.93 (0.85-1.02)	0.92 (0.84-1.01)	
**Drinker**					
No	1.00 (ref)	0.95 (0.79-1.13)	1.01 (0.85-1.20)	1.11 (0.93-1.32)	0.287
Yes	1.00 (ref)	1.02 (0.93-1.12)	0.95 (0.87-1.05)	0.92 (0.83-1.01)	
**Smoking status**					
Never	1.00 (ref)	0.96 (0.86-1.08)	0.94 (0.84-1.05)	0.95 (0.85-1.07)	
Previous	1.00 (ref)	1.04 (0.91-1.18)	1.01 (0.89-1.15)	0.96 (0.84-1.10)	0.821
Current	1.00 (ref)	1.13 (0.83-1.52)	0.92 (0.68-1.26)	1.05 (0.77-1.44)	
**Physical activity**					
Low	1.00 (ref)	0.94 (0.83-1.06)	0.93 (0.82-1.06)	0.91 (0.80-1.04)	
Moderate	1.00 (ref)	1.11 (0.96-1.28)	1.06 (0.92-1.22)	1.05 (0.90-1.21)	0.213
High	1.00 (ref)	1.00 (0.86-1.18)	0.93 (0.80-1.10)	0.94 (0.80-1.10)	

Estimated effects were calculated using Cox model stratified by age, sex, UK Biobank assessment centers, and additionally adjusted for overall health rating, long-standing illness, education, index of multiple deprivation, multivitamin use, mineral use, smoking status, alcohol consumption, physical activity and body mass index.

Supplementary Table S20. Subgroup analysis for the association between uPDI and risk of cholelithiasis incidence.

Subgroups	HR (95% CI)				*P* for interaction
	Q1	Q2	Q3	Q4	
**Age**					
<60 years	1.00 (ref)	1.12 (0.99-1.27)	1.04 (0.92-1.18)	1.09 (0.96-1.23)	0.249
≥60 years	1.00 (ref)	0.99 (0.88-1.12)	1.10 (0.98-1.23)	1.08 (0.96-1.22)	
**Gender**					
Female	1.00 (ref)	1.05 (0.95-1.17)	1.00 (0.90-1.11)	1.05 (0.94-1.17)	0.173
Male	1.00 (ref)	1.06 (0.92-1.23)	1.18 (1.03-1.36)	1.13 (0.98-1.30)	
**Education level**					
College or University degree	1.00 (ref)	1.02 (0.88-1.19)	1.05 (0.91-1.22)	1.06 (0.91-1.23)	0.979
Other	1.00 (ref)	1.06 (0.96-1.18)	1.07 (0.97-1.18)	1.08 (0.97-1.20)	
**Body mass index**					
<25 kg/m^2	1.00 (ref)	1.18 (0.99-1.41)	1.16 (0.96-1.38)	1.19 (0.99-1.44)	0.127
≥25 kg/m^2	1.00 (ref)	1.02 (0.93-1.13)	1.06 (0.97-1.17)	1.10 (1.00-1.21)	
**Drinker**					
No	1.00 (ref)	0.98 (0.82-1.16)	0.87 (0.73-1.04)	0.88 (0.74-1.05)	0.131
Yes	1.00 (ref)	1.08 (0.98-1.20)	1.13 (1.03-1.25)	1.15 (1.04-1.26)	
**Smoking status**					
Never	1.00 (ref)	0.99 (0.88-1.11)	1.01 (0.90-1.13)	1.05 (0.94-1.18)	
Previous	1.00 (ref)	1.15 (1.00-1.31)	1.13 (0.99-1.30)	1.11 (0.97-1.27)	0.773
Current	1.00 (ref)	1.00 (0.73-1.38)	1.07 (0.78-1.46)	1.03 (0.74-1.42)	
**Physical activity**					
Low	1.00 (ref)	1.07 (0.94-1.23)	1.05 (0.92-1.20)	1.12 (0.98-1.28)	
Moderate	1.00 (ref)	1.10 (0.95-1.27)	1.08 (0.94-1.25)	1.03 (0.89-1.20)	0.424
High	1.00 (ref)	0.99 (0.85-1.16)	1.07 (0.92-1.25)	1.07 (0.91-1.25)	

Estimated effects were calculated using Cox model stratified by age, sex, UK Biobank assessment centers, and additionally adjusted for overall health rating, long-standing illness, education, index of multiple deprivation, multivitamin use, mineral use, smoking status, alcohol consumption, physical activity and body mass index.

Supplementary Table S21. Subgroup analysis for the association between HEI-2015 and risk of cholelithiasis incidence.

Subgroups	HR (95% CI)				*P* for interaction
	Q1	Q2	Q3	Q4	
**Age**					
<60 years	1.00 (ref)	1.00 (0.90-1.11)	0.92 (0.83-1.03)	0.83 (0.74-0.94)	0.360
≥60 years	1.00 (ref)	1.08 (0.96-1.21)	0.98 (0.88-1.10)	0.95 (0.85-1.07)	
**Gender**					
Female	1.00 (ref)	1.05 (0.95-1.16)	1.03 (0.93-1.15)	0.93 (0.84-1.03)	0.173
Male	1.00 (ref)	1.02 (0.91-1.15)	0.84 (0.74-0.95)	0.85 (0.74-0.97)	
**Education level**					
College or University degree	1.00 (ref)	1.03 (0.89-1.20)	0.92 (0.80-1.07)	0.92 (0.80-1.07)	0.391
Other	1.00 (ref)	1.03 (0.94-1.12)	0.96 (0.87-1.05)	0.86 (0.78-0.95)	
**Body mass index**					
<25 kg/m^2	1.00 (ref)	1.03 (0.87-1.23)	0.89 (0.74-1.06)	0.83 (0.69-0.99)	0.045
≥25 kg/m^2	1.00 (ref)	1.04 (0.96-1.14)	0.98 (0.89-1.07)	0.91 (0.83-1.00)	
**Drinker**					
No	1.00 (ref)	1.05 (0.89-1.23)	1.00 (0.84-1.18)	0.77 (0.65-0.92)	0.202
Yes	1.00 (ref)	1.02 (0.94-1.12)	0.94 (0.86-1.03)	0.93 (0.85-1.02)	
**Smoking status**					
Never	1.00 (ref)	1.08 (0.97-1.20)	0.99 (0.89-1.11)	0.91 (0.82-1.02)	
Previous	1.00 (ref)	1.02 (0.90-1.15)	0.93 (0.82-1.06)	0.91 (0.80-1.03)	0.519
Current	1.00 (ref)	0.88 (0.68-1.15)	0.79 (0.59-1.06)	0.71 (0.50-0.99)	
**Physical activity**					
Low	1.00 (ref)	1.01 (0.89-1.13)	1.00 (0.89-1.13)	0.89 (0.78-1.01)	
Moderate	1.00 (ref)	0.99 (0.86-1.13)	0.88 (0.77-1.01)	0.86 (0.75-0.99)	0.797
High	1.00 (ref)	1.13 (0.97-1.31)	0.96 (0.82-1.12)	0.91 (0.78-1.07)	

Estimated effects were calculated using Cox model stratified by age, sex, UK Biobank assessment centers, and additionally adjusted for overall health rating, long-standing illness, education, index of multiple deprivation, multivitamin use, mineral use, smoking status, alcohol consumption, physical activity and body mass index.

Supplementary Table S22. Subgroup analysis for the association between EAT-lancet score and risk of cholelithiasis incidence.

Subgroups	HR (95% CI)				*P* for interaction
	Q1	Q2	Q3	Q4	
**Age**					
<60 years	1.00 (ref)	1.02 (0.90-1.16)	1.09 (0.97-1.23)	1.04 (0.92-1.17)	0.090
≥60 years	1.00 (ref)	0.92 (0.82-1.04)	0.91 (0.81-1.02)	0.96 (0.86-1.08)	
**Gender**					
Female	1.00 (ref)	0.92 (0.82-1.03)	0.95 (0.85-1.06)	0.95 (0.85-1.06)	0.438
Male	1.00 (ref)	1.03 (0.90-1.17)	1.06 (0.93-1.21)	1.06 (0.93-1.21)	
**Education level**					
College or University degree	1.00 (ref)	0.89 (0.75-1.04)	1.05 (0.90-1.22)	1.03 (0.89-1.20)	0.2256
Other	1.00 (ref)	1.00 (0.91-1.11)	0.98 (0.88-1.08)	0.98 (0.89-1.09)	
**Body mass index**					
<25 kg/m^2	1.00 (ref)	1.04 (0.85-1.27)	0.96 (0.79-1.17)	0.98 (0.81-1.19)	0.406
≥25 kg/m^2	1.00 (ref)	0.94 (0.85-1.03)	0.99 (0.90-1.08)	0.97 (0.89-1.07)	
**Drinker**					
No	1.00 (ref)	0.93 (0.77-1.12)	0.97 (0.81-1.16)	1.05 (0.88-1.25)	0.279
Yes	1.00 (ref)	0.98 (0.89-1.08)	1.00 (0.91-1.10)	0.98 (0.89-1.08)	
**Smoking status**					
Never	1.00 (ref)	0.95 (0.84-1.07)	0.97 (0.87-1.09)	0.95 (0.85-1.07)	
Previous	1.00 (ref)	0.99 (0.86-1.13)	1.04 (0.91-1.19)	1.07 (0.94-1.23)	0.859
Current	1.00 (ref)	1.01 (0.74-1.38)	0.98 (0.72-1.33)	0.99 (0.73-1.35)	
**Physical activity**					
Low	1.00 (ref)	1.04 (0.90-1.19)	1.08 (0.95-1.24)	1.01 (0.88-1.16)	
Moderate	1.00 (ref)	0.93 (0.80-1.09)	1.03 (0.89-1.19)	1.01 (0.88-1.17)	0.198
High	1.00 (ref)	0.92 (0.78-1.09)	0.84 (0.72-1.00)	0.95 (0.82-1.12)	

Estimated effects were calculated using Cox model stratified by age, sex, UK Biobank assessment centers, and additionally adjusted for overall health rating, long-standing illness, education, index of multiple deprivation, multivitamin use, mineral use, smoking status, alcohol consumption, physical activity and body mass index.

Supplementary Table S23. Sensitivity analyses of the cholelithiasis risk according to MED and the level of genetic risk[a].

Group	Sensitivity analysis[b]		Sensitivity analysis[c]		Sensitivity analysis[d]	
	**HR (95%CI)**	**P value**	**HR (95%CI)**	**P value**	**HR (95%CI)**	**P value**
**High genetic risk**						
Quartile 1	1.00 (ref)		1.00 (ref)		1.00 (ref)	
Quartile 4	1.07 (0.85-1.36)	0.564	1.06 (0.83-1.34)	0.646	1.07 (0.85-1.33)	0.574
**Intermediate genetic risk**						
Quartile 1	1.02 (0.79-1.30)	0.899	1.00 (0.78-1.29)	0.982	0.98 (0.78-1.25)	0.898
Quartile 4	0.94 (0.75-1.18)	0.601	0.92 (0.73-1.15)	0.450	0.92 (0.75-1.14)	0.470
**Low genetic risk**						
Quartile 1	0.92 (0.67-1.26)	0.597	0.90 (0.65-1.23)	0.492	0.92 (0.69-1.25)	0.604
Quartile 4	0.90 (0.71-1.15)	0.396	0.87 (0.69-1.11)	0.273	0.91 (0.73-1.15)	0.442

[a] Hazard ratio and 95% CI were estimated from multivariate Cox regression models stratified by age, sex, UK Biobank assessment centers, and additionally adjusted for overall health rating, long-standing illness, education, index of multiple deprivation, multivitamin use, mineral use, smoking status, alcohol consumption, physical activity and body mass index.
[b] Sensitivity analysis lagged the exposure by 2 years.
[c] Sensitivity analysis lagged the exposure by 4 years.
[d] sensitivity analysis removed the variable BMI from the multivariable-adjusted model.

Supplementary Table S24. Sensitivity analyses of the cholelithiasis risk according to aMED and the level of genetic risk[a].

	Sensitivity analysis[b]		Sensitivity analysis[c]		Sensitivity analysis[d]	
**Group**	HR (95%CI)	P value	HR (95%CI)	P value	HR (95%CI)	P value
**High genetic risk**						
Quartile 1	1.00 (ref)		1.00 (ref)		1.00 (ref)	
Quartile 4	0.96 (0.81-1.13)	0.604	0.95 (0.80-1.13)	0.568	0.95 (0.81-1.12)	0.538
**Intermediate genetic risk**						
Quartile 1	0.93 (0.80-1.07)	0.321	0.93 (0.80-1.07)	0.311	0.92 (0.80-1.06)	0.269
Quartile 4	0.78 (0.68-0.91)	0.001	0.77 (0.67-0.89)	0.001	0.76 (0.66-0.88)	<0.001
**Low genetic risk**						
Quartile 1	0.75 (0.62-0.91)	0.004	0.75 (0.62-0.91)	0.003	0.79 (0.66-0.95)	0.012
Quartile 4	0.74 (0.62-0.89)	0.001	0.73 (0.61-0.88)	0.001	0.74 (0.63-0.88)	0.001

[a] Hazard ratio and 95% CI were estimated from multivariate Cox regression models stratified by age, sex, UK Biobank assessment centers, and additionally adjusted for overall health rating, long-standing illness, education, index of multiple deprivation, multivitamin use, mineral use, smoking status, alcohol consumption, physical activity and body mass index.
[b] Sensitivity analysis lagged the exposure by 2 years.
[c] Sensitivity analysis lagged the exposure by 4 years.
[d] sensitivity analysis removed the variable BMI from the multivariable-adjusted model.

Supplementary Table S25. Sensitivity analyses of the cholelithiasis risk according to PDI and the level of genetic risk[a].

	Sensitivity analysis[b]		Sensitivity analysis[c]		Sensitivity analysis[d]	
**Group**	**HR (95%CI)**	**P value**	**HR (95%CI)**	**P value**	**HR (95%CI)**	**P value**
**High genetic risk**						
Quartile 1	1.00 (ref)		1.00 (ref)		1.00 (ref)	
Quartile 4	0.93 (0.78-1.10)	0.398	0.94 (0.78-1.11)	0.452	0.86 (0.73-1.02)	0.082
**Intermediate genetic risk**						
Quartile 1	0.84 (0.73-0.97)	0.021	0.85 (0.73-0.98)	0.028	0.82 (0.72-0.94)	0.004
Quartile 4	0.88 (0.76-1.01)	0.068	0.88 (0.76-1.01)	0.074	0.81 (0.71-0.93)	0.002
**Low genetic risk**						
Quartile 1	0.80(0.67-0.96)	0.016	0.80 (0.67-0.97)	0.02	0.80 (0.67-0.95)	0.012
Quartile 4	0.82 (0.68-0.98)	0.028	0.81 (0.67-0.97)	0.023	0.78 (0.66-0.92)	0.004

[a] Hazard ratio and 95% CI were estimated from multivariate Cox regression models stratified by age, sex, UK Biobank assessment centers, and additionally adjusted for overall health rating, long-standing illness, education, index of multiple deprivation, multivitamin use, mineral use, smoking status, alcohol consumption, physical activity and body mass index.
[b] Sensitivity analysis lagged the exposure by 2 years.
[c] Sensitivity analysis lagged the exposure by 4 years.
[d] sensitivity analysis removed the variable BMI from the multivariable-adjusted model.

Supplementary Table S26. Sensitivity analyses of the cholelithiasis risk according to hPDI and the level of genetic risk[a].

	Sensitivity analysis[b]		Sensitivity analysis[c]		Sensitivity analysis[d]	
**Group**	**HR (95%CI)**	**P value**	**HR (95%CI)**	**P value**	**HR (95%CI)**	**P value**
**High genetic risk**						
Quartile 1	1.00 (ref)		1.00 (ref)		1.00 (ref)	
Quartile 4	1.03 (0.86-1.24)	0.713	1.03 (0.86-1.24)	0.718	0.87 (0.73-1.04)	0.116
**Intermediate genetic risk**						
Quartile 1	0.91 (0.78-1.06)	0.224	0.91 (0.78-1.07)	0.266	0.85 (0.73-0.98)	0.026
Quartile 4	0.88 (0.76-1.03)	0.113	0.89 (0.76-1.04)	0.129	0.75 (0.65-0.87)	<0.001
**Low genetic risk**						
Quartile 1	0.88 (0.72-1.07)	0.190	0.87 (0.72-1.07)	0.186	0.84 (0.70-1.01)	0.062
Quartile 4	0.81 (0.67-0.99)	0.036	0.79 (0.65-0.97)	0.023	0.73 (0.61-0.87)	0.001

[a] Hazard ratio and 95% CI were estimated from multivariate Cox regression models stratified by age, sex, UK Biobank assessment centers, and additionally adjusted for overall health rating, long-standing illness, education, index of multiple deprivation, multivitamin use, mineral use, smoking status, alcohol consumption, physical activity and body mass index.
[b] Sensitivity analysis lagged the exposure by 2 years.
[c] Sensitivity analysis lagged the exposure by 4 years.
[d] sensitivity analysis removed the variable BMI from the multivariable-adjusted model.

Supplementary Table S27. Sensitivity analyses of the cholelithiasis risk according to uPDI and the level of genetic risk[a].

Group	Sensitivity analysis[b]		Sensitivity analysis[c]		Sensitivity analysis[d]	
	**HR (95%CI)**	**P value**	**HR (95%CI)**	**P value**	**HR (95%CI)**	**P value**
**High genetic risk**						
Quartile 4	1.00 (ref)		1.00 (ref)		1.00 (ref)	
Quartile 1	1.04 (0.87-1.25)	0.650	1.03 (0.86-1.25)	0.720	0.89 (0.75-1.06)	0.199
**Intermediate genetic risk**						
Quartile 4	0.93 (0.80-1.07)	0.292	0.93 (0.80-1.07)	0.295	0.87 (0.76-1.00)	0.047
Quartile 1	0.86 (0.74-1.00)	0.047	0.86 (0.74-1.00)	0.045	0.75 (0.65-0.86)	<0.001
**Low genetic risk**						
Quartile 4	0.91 (0.76-1.08)	0.274	0.90 (0.75-1.08)	0.251	0.88 (0.75-1.05)	0.149
Quartile 1	0.79 (0.64-0.96)	0.021	0.77 (0.62-0.94)	0.011	0.73 (0.60-0.88)	0.001

[a] Hazard ratio and 95% CI were estimated from multivariate Cox regression models stratified by age, sex, UK Biobank assessment centers, and additionally adjusted for overall health rating, long-standing illness, education, index of multiple deprivation, multivitamin use, mineral use, smoking status, alcohol consumption, physical activity and body mass index.
[b] Sensitivity analysis lagged the exposure by 2 years.
[c] Sensitivity analysis lagged the exposure by 4 years.
[d] sensitivity analysis removed the variable BMI from the multivariable-adjusted model.

Supplementary Table S28. Sensitivity analyses of the cholelithiasis risk according to HEI-2015 and the level of genetic risk[a].

	Sensitivity analysis[b]		Sensitivity analysis[c]		Sensitivity analysis[d]	
**Group**	**HR (95%CI)**	**P value**	**HR (95%CI)**	**P value**	**HR (95%CI)**	**P value**
**High genetic risk**						
Quartile 1	1.00 (ref)		1.00 (ref)		1.00 (ref)	
Quartile 4	0.84 (0.70-1.00)	0.051	0.84 (0.70-1.00)	0.053	0.89 (0.75-1.06)	0.185
**Intermediate genetic risk**						
Quartile 1	0.83 (0.72-0.95)	0.008	0.82 (0.72-0.95)	0.007	0.83 (0.73-0.95)	0.007
Quartile 4	0.73 (0.64-0.85)	<0.001	0.73 (0.63-0.84)	<0.001	0.74 (0.65-0.85)	<0.001
**Low genetic risk**						
Quartile 1	0.82 (0.69-0.98)	0.025	0.81 (0.68-0.97)	0.020	0.87 (0.74-1.03)	0.102
Quartile 4	0.70 (0.58-0.85)	<0.001	0.70 (0.58-0.85)	<0.001	0.73 (0.61-0.87)	0.001

[a] Hazard ratio and 95% CI were estimated from multivariate Cox regression models stratified by age, sex, UK Biobank assessment centers, and additionally adjusted for overall health rating, long-standing illness, education, index of multiple deprivation, multivitamin use, mineral use, smoking status, alcohol consumption, physical activity and body mass index.
[b] Sensitivity analysis lagged the exposure by 2 years.
[c] Sensitivity analysis lagged the exposure by 4 years.
[d] sensitivity analysis removed the variable BMI from the multivariable-adjusted model.

Supplementary Table S29. Sensitivity analyses of the cholelithiasis risk according to EAT-lancet score and the level of genetic risk[a].

Group	Sensitivity analysis[b]		Sensitivity analysis[c]		Sensitivity analysis[d]	
	**HR (95%CI)**	**P value**	**HR (95%CI)**	**P value**	**HR (95%CI)**	**P value**
**High genetic risk**						
Quartile 1	1.00 (ref)		1.00 (ref)		1.00 (ref)	
Quartile 4	1.02 (0.85-1.23)	0.813	1.04 (0.86-1.25)	0.723	0.94 (0.79-1.13)	0.526
**Intermediate genetic risk**						
Quartile 1	0.96 (0.81-1.14)	0.653	0.98 (0.82-1.16)	0.794	0.93 (0.79-1.09)	0.354
Quartile 4	0.94 (0.79-1.10)	0.434	0.93 (0.79-1.10)	0.426	0.85 (0.73-1.00)	0.047
**Low genetic risk**						
Quartile 1	0.86 (0.69-1.07)	0.176	0.85 (0.68-1.06)	0.148	0.84 (0.68-1.03)	0.100
Quartile 4	0.90 (0.74-1.10)	0.312	0.89 (0.73-1.09)	0.266	0.85 (0.71-1.02)	0.085

[a] Hazard ratio and 95% CI were estimated from multivariate Cox regression models stratified by age, sex, UK Biobank assessment centers, and additionally adjusted for overall health rating, long-standing illness, education, index of multiple deprivation, multivitamin use, mineral use, smoking status, alcohol consumption, physical activity and body mass index.
[b] Sensitivity analysis lagged the exposure by 2 years.
[c] Sensitivity analysis lagged the exposure by 4 years.
[d] sensitivity analysis removed the variable BMI from the multivariable-adjusted model.

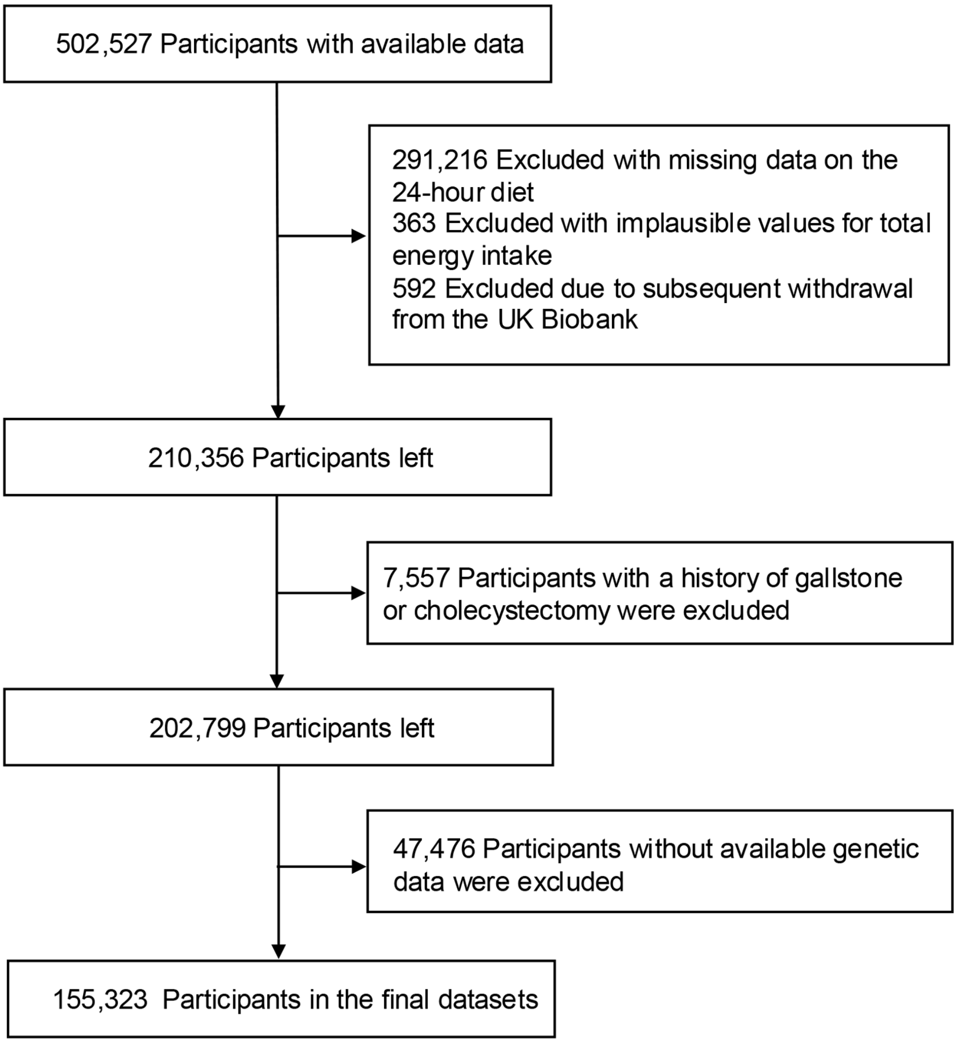

Supplementary Figure S1. Flow diagram of participants in the present study.

A

Characteristics	Cases/ Person-years	HR (95% CI)	HR (95% CI)	*P* value
**High genetic risk**				
Quartile 1	97 / 36523.83		1.00 (ref)	
Quartile 4	509 / 196064.89		1.04 (0.83-1.31)	0.709
**Intermediate genetic risk**				
Quartile 1	276 / 109524.09		0.96 (0.75-1.22)	0.723
Quartile 4	1345 / 595488.55		0.92 (0.74-1.14)	0.451
**Low genetic risk**				
Quartile 1	84 / 36191.09		0.90 (0.67-1.21)	0.482
Quartile 4	436 / 194788.18		0.91 (0.72-1.14)	0.395
		0.5 1.0 1.5		

B

Characteristics	Cases/ Person-years	HR (95% CI)	HR (95% CI)	*P* value
**High genetic risk**				
Quartile 1	299 / 95190.88		1.00 (ref)	
Quartile 4	285 / 116516.12		0.91 (0.77-1.07)	0.257
**Intermediate genetic risk**				
Quartile 1	735 / 288245.86		0.82 (0.72-0.94)	0.005
Quartile 4	818 / 350617.7		0.86 (0.75-0.99)	0.034
**Low genetic risk**				
Quartile 1	237 / 93335.88		0.81 (0.68-0.96)	0.015
Quartile 4	257 / 115383.59		0.82 (0.69-0.97)	0.022
		0.5 1.0		

C

Characteristics	Cases/ Person-years	HR (95% CI)	HR (95% CI)	*P* value
**High genetic risk**				
Quartile 1	255 / 86822.2		1.00 (ref)	
Quartile 4	272 / 103731.27		0.96 (0.81-1.15)	0.667
**Intermediate genetic risk**				
Quartile 1	666 / 261236.28		0.86 (0.74-1.00)	0.048
Quartile 4	724 / 315487.14		0.83 (0.72-0.96)	0.013
**Low genetic risk**				
Quartile 1	213 / 85306.64		0.85 (0.71-1.03)	0.091
Quartile 4	221 / 102335.57		0.80 (0.67-0.96)	0.018
		0.5 1.0		

D

Characteristics	Cases/ Person-years	HR (95% CI)	HR (95% CI)	*P* value
**High genetic risk**				
Quartile 4	298 / 104139.72		1.00 (ref)	
Quartile 1	223 / 85572.01		0.98 (0.82-1.17)	0.826
**Intermediate genetic risk**				
Quartile 4	802 / 314829.82		0.89 (0.78-1.02)	0.087
Quartile 1	574 / 260220.91		0.82 (0.71-0.94)	0.006
**Low genetic risk**				
Quartile 4	261 / 101516.07		0.9 (0.76-1.06)	0.211
Quartile 1	176 / 83739.13		0.8 (0.66-0.97)	0.021
		0.5 1.0		

E

Characteristics	Cases/ Person-years	HR (95% CI)	HR (95% CI)	*P* value
**High genetic risk**				
Quartile 1	202 / 70821.98		1.00 (ref)	
Quartile 4	332 / 127124.33		0.99 (0.83-1.19)	0.928
**Intermediate genetic risk**				
Quartile 1	571 / 217750.93		0.92 (0.78-1.08)	0.178
Quartile 4	883 / 381024.7		0.90 (0.77-1.05)	0.100
**Low genetic risk**				
Quartile 1	170 / 71403.19		0.84 (0.68-1.03)	0.092
Quartile 4	287 / 123743.74		0.89 (0.74-1.07)	0.222
		0.5 1.0		

Supplementary Figure S2. Joint effects and interactions of dietary patterns and PRS on cholelithiasis risk. (A) MED (B) PDI (C) hPDI (D) uPDI (E) EAT-lancet score.

Estimated effects were calculated using Cox model stratified by age, sex, UK Biobank assessment centers, and additionally adjusted for overall health rating, long-standing illness, education, index of multiple deprivation, multivitamin use, mineral use, smoking status, alcohol consumption, physical activity and body mass index. Abbreviations: ref = reference, HR: Hazard Ratio, CI, CI: Confidence Interval.
